# A Systematic Review and Meta-Analysis of Social Cognition Among People Living with HIV: Implications for Non-Social Cognition and Social Everyday Functioning

**DOI:** 10.1007/s11065-024-09643-5

**Published:** 2024-06-13

**Authors:** David E. Vance, Rebecca Billings, Crystal Chapman Lambert, Pariya L. Fazeli, Burel R. Goodin, Mirjam-Colette Kempf, Leah H. Rubin, Bulent Turan, Jenni Wise, Gerhard Hellemann, Junghee Lee

**Affiliations:** 1 School of Nursing, University of Alabama at Birmingham, Birmingham, AL USA; 2 UAB Libraries, University of Alabama at Birmingham, Birmingham, AL USA; 3 Department of Anesthesiology, Washington University Pain Center, Washington University, St. Louis, MO USA; 4 Department of Neurology, School of Medicine, Johns Hopkins University, Baltimore, MD USA; 5 Department of Psychology, Koc University, Rumelifeneri Caddesi, Turkey; 6 Department of Biostatistics, School of Public Health, University of Alabama at Birmingham, Birmingham, AL USA; 7 Department of Psychiatry and Behavioral Neurobiology, Heersink School of Medicine, University of Alabama at Birmingham, Birmingham, AL USA

**Keywords:** Social cognition, HIV, Emotional processing, Stigma, Theory of mind

## Abstract

Social cognition—the complex mental ability to perceive social stimuli and negotiate the social environment—has emerged as an important cognitive ability needed for social functioning, everyday functioning, and quality of life. Deficits in social cognition have been well documented in those with severe mental illness including schizophrenia and depression, those along the autism spectrum, and those with other brain disorders where such deficits profoundly impact everyday life. Moreover, subtle deficits in social cognition have been observed in other clinical populations, especially those that may have compromised non-social cognition (i.e., fluid intelligence such as memory). Among people living with HIV (PLHIV), 44% experience cognitive impairment; likewise, social cognitive deficits in theory of mind, prosody, empathy, and emotional face recognition/perception are gradually being recognized. This systematic review and meta-analysis aim to summarize the current knowledge of social cognitive ability among PLHIV, identified by 14 studies focused on social cognition among PLHIV, and provides an objective consensus of the findings. In general, the literature suggests that PLHIV may be at-risk of developing subtle social cognitive deficits that may impact their everyday social functioning and quality of life. The causes of such social cognitive deficits remain unclear, but perhaps develop due to (1) HIV-related sequelae that are damaging the same neurological systems in which social cognition and non-social cognition are processed; (2) stress related to coping with HIV disease itself that overwhelms one’s social cognitive resources; or (3) may have been present pre-morbidly, possibly contributing to an HIV infection. From this, a theoretical framework is proposed highlighting the relationships between social cognition, non-social cognition, and social everyday functioning.

**Supplementary Information:**

The online version contains supplementary material available at 10.1007/s11065-024-09643-5.

## Introduction

Despite treatment advances making HIV disease a manageable chronic health condition, HIV remains highly distressing and can compromise one’s access to healthcare, social interactions, and quality of life (Turan et al., 2019; Yuvaraj et al., 2020). The preponderance of studies show that social support remains important in buffering against the negative effects of HIV among people living with HIV (PLHIV) (Rzeszutek, 2018; Slater et al., 2013). Yet, an ability that may be pivotal in negotiating the social environment to cultivate such social support is social cognition.

Social cognition—the complex mental ability to perceive, understand, and respond to socially relevant stimuli and navigate the social environment—has emerged as an important ability that subserves efficient social functioning and garners social support in everyday life (Adolphs, 2001). Social cognition is vital to social everyday functioning, from interacting with strangers on the street, making friends and social supports, to maintaining intimate relationships, to bargaining socially and financially, interacting with employees, and detecting threats in one’s social environment. If one is not able to accurately interpret social cues (e.g., facial expression) or others’ mental states (e.g., emotions, intentions, thoughts), especially in a complex social environment surrounded by HIV and within the context of medical care, this can create numerous issues in misunderstanding people, resulting in poor outcomes to the individual. Consistent with this view, a large body of work in clinical psychology and neurological disorders showed that people with schizophrenia (Fett et al., 2011; Green et al., 2015) and autism spectrum disorders (Velikonja et al., 2019), both of which are characterized with marked social dysfunction, demonstrate social cognitive impairments that profoundly impact everyday life. Further, more subtle deficits in social cognition have also been observed in other clinical populations (e.g., adults with mild cognitive impairment; alcohol use disorder) which can also exert difficulty in social relationships that impair social everyday functioning and quality of life (Bora & Yener, 2017; Le Berre, 2019). Below, we briefly present representative social cognitive constructs relevant to this discussion, specifically in emotional face recognition/perception, prosody perception, theory of mind, and empathy.

Facial emotional recognition/perception (i.e., affective face perception) is the most studied social cognitive construct both in social neuroscience and clinical neuroscience (Beaudoin & Beauchamp, 2020). Non-affective face perception involves the perception of structural cues from facial stimuli (e.g., discriminating older faces from younger faces, selecting female faces from male faces). Affective face perception involves the perception of affective information garnered from facial stimuli. Affective face perception can be assessed directly or indirectly. Typical tasks that indirectly measure affective face perception ask participants to discriminate older from younger faces or female from male faces while presenting faces with emotional expressions. In this way, emotional expression of face stimuli is not relevant to the task goal, but its effect on performance can be assessed. Typical tasks that directly assess affective face perception ask participants to perceive emotional quality of faces (e.g., happy, sad, angry). Most paradigms on affective face perception primarily employed static (i.e., not moving) face stimuli that show canonical facial expression, rather than dynamic facial stimuli that express subtle and changing emotions, like facial expressions that are typically encountered in everyday life. Emerging studies suggest that PLHIV may experience some mild impairment in emotional facial recognition (Clark et al., 2010, 2015); if so, this could impair their ability to negotiate their social environment, an environment where some may misperceive threat or acceptance.

Prosody perception involves an ability to perceive emotions that are conveyed through acoustic properties of voices (e.g., pitch, intonation) (Jasmin et al., 2021). Like affective face perception, prosody perception can be assessed indirectly or directly by either making emotional quality of voices task-relevant or not. Within the context of HIV, emerging studies suggest PLHIV also experience poorer prosody compared to controls (González-Baeza et al., 2017).

Theory of mind refers to an ability to take perspectives of other people and infer their mental states, such as belief, thought, or intention (Beaudoin & Beauchamp, 2020; Carlson et al., 2013). Theory of mind is also known as mentalizing or mental state attribution. Theory of mind can be assessed with a range of stimuli including cartoons, vignettes, or videos. After being presented with these stimuli, a participant is asked to infer belief or intention of another person whose perspective may or may not differ from the perspective of the participant. Within the context of HIV, preliminary evidence suggests that PLHIV may exhibit comparable impairments in this ability as those with schizophrenia (Lysaker et al., 2012). Also, such poorer theory of mind ability has been linked to more inclination to engage in risky health behaviors (Walzer et al., 2016).

Empathy broadly refers to an ability to share and understand emotional states of other people and includes affect sharing and empathic accuracy (Beaudoin & Beauchamp, 2020; Decety, 2010). Affect sharing involves an ability to share emotional states of another person (e.g., feeling pain when looking at a picture depicting a person being hurt in a car accident) and typically does not involve inferential processes. To the contrary, empathic accuracy involves an ability to accurately infer emotional states of other people. A typical paradigm on empathic accuracy asks a participant to continuously rate the emotional states of another person (i.e., a target) while watching a brief clip that depicts a target describing an autobiographical event and assesses empathic accuracy by examining the correspondence between a participant’s rating and a target’s own rating (Lee et al., 2011). In addition to these paradigms, self-report questionnaires have also been used to assess the subjective evaluation of empathic experiences. For instance, the Interpersonal Reactivity Index (IRI) (Davis, 1980) is composed of four subscales, each assessing different facets of empathy. Within the context of HIV, very little is known about empathy, but what little is known suggests a link between empathy and the propensity to engage less in risky health behaviors (Walzer et al., 2016).

Deficits in social cognition are gradually being recognized in PLHIV (Baldonero et al., 2013; Grabyan et al., 2018). The reasons for the development of such deficits are unclear, but perhaps they are due to (1) compromised non-social cognitive functioning, that is fluid cognitive abilities (i.e., executive function, memory), common in PLHIV that impact the same neurological systems in which social cognition is processed, or (2) stress related to coping with the HIV infection itself that overwhelms one’s social cognitive resources. In fact, it is conceivable that some PLWH may have had premorbid social cognitive deficits that may have contributed to their HIV infection. The purpose of this systematic review and meta-analysis of social cognition in the context of HIV was to examine and summarize the existing literature and provide an objective consensus of the findings. A synthesis of the findings and an examination of the methodology was provided to recommend the next steps to propel the science in this area. Based on the results of this systematic review and meta-analysis, a rudimentary theoretical framework has been proposed in which to enhance our understanding of how social cognition relates to non-social cognition and social everyday functioning, with an emphasis on how it fits into the larger HIV literature. Implications for practice and research are posited.

## Systematic Review of Social Cognition in the Context of HIV Infection

### Systematic Review Methodology

**Fig. 1:**
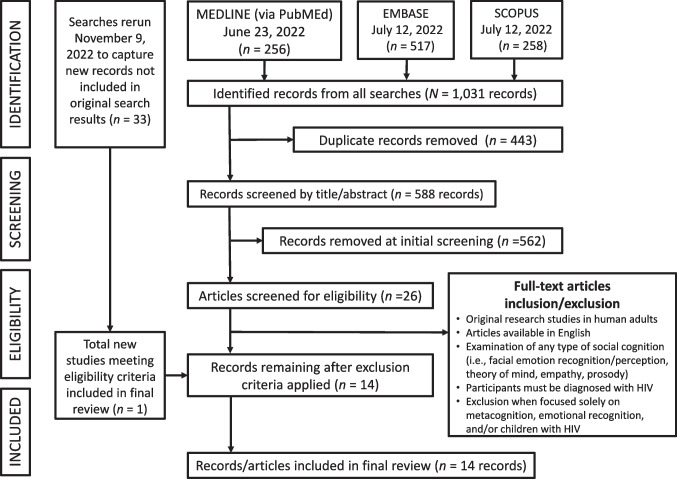
PRISMA diagram demonstrating search and screening method for HIV social cognition literature

Using the Preferred Reporting Items for Systematic Reviews and Meta-Analyses (PRISMA) approach (Moher et al., 2009), MEDLINE (via PubMed) on June 23, 2022, and Embase and Scopus databases on July 12, 2022, were searched for research studies on social cognition in PLHIV (Fig.1). Full search strategies and terms from each database (e.g., theory of mind) are provided in Table 1. From this, 1031 records were identified, and 443 duplicates were removed, leaving 588 records to be reviewed. As it took us time to process and summarize these data, the search was rerun in the three databases on November 9, 2022, to capture any new publications that had been published since the original search date. A total of 33 new references were captured and added to Covidence for screening. Using the Covidence software, two of the authors (JL & DEV) reviewed these article titles, abstracts, and articles separately to determine whether the article met study inclusion criteria. More precisely, the articles (in English) were evaluated for the following inclusion criteria: (1) original research studies in adult humans (not systematic reviews, review articles, or case reports); (2) examination of any social cognition construct using performance tasks (i.e., emotional face recognition/perception, prosody, theory of mind, and empathy) and/or survey; and (3) participant sample must include PLHIV. Studies not meeting these inclusion criteria were excluded. More specific exclusion criteria included (1) focus entirely on emotional regulation without using performance tasks or survey; (2) focus entirely on metacognition; and/or (3) focus on children living with HIV. We (two of the authors who reviewed each article independently) compared their findings of the selected studies and discussed each one until a consensus was met on whether the article met the criteria. In evaluating which studies met criteria, some studies may appear to meet the eligibility criteria, but on further examination, the elements needed for this systematic review were not found. For example, in an article by Schulte et al. (2011), researchers were examining disruption of emotion and conflict processing in HIV infection with and without alcoholism comorbidity. These researchers were using emotionally salient words and faces to examine how this disrupts cognitive functioning. Although emotionally salient faces were used, emotional face recognition/perception was not being examined or measured; thus, such studies like this were excluded. For studies that did meet the eligibility criteria, data items were extract from the articles that met criteria; data items entailed the study characteristics (i.e., sample size, sample characteristics, design), instrument used to measure a particular social cognitive ability, related cognitive findings, major findings, and study strengths/limitations as detailed in Tables 2 and 3. These tables served as an effective method to collect and summarize the data by being able to tabulate and examining findings.

**Table 1:** Terms used to search for articles in PubMed, Embase, and Scopus

**Database**	**Search Step**	**Searches**	**Results**
**Pubmed** Host: National Library of Medicine Data Parameters: 1946 to Present Date Searched: Date: 06/23/2022 & 11/09/2022 Searcher: Becca Billings	1	(“facial affect perception”[tiab] OR “facial affect recognition”[tiab] OR “prosody”[tiab] OR “affect perception”[tiab] OR “affect recognition”[tiab] OR “emotion regulation”[tiab] OR “theory of mind”[tiab] OR “mental state attribution”[tiab] OR “affect sharing”[tiab] OR “empathy”[tiab] OR “affect empathy”[tiab] OR “cognitive empathy”[tiab] OR “self-other processing”[tiab] OR “self-referential processing”[tiab] OR “attribution bias”[tiab] OR “mirror neuron”[tiab] OR “social cognition”[tiab] OR “emotion processing”[tiab] OR “facial emotion*”[tiab])	46,030
	2	(emotion*[tiab] AND cognit*[tiab] AND process*[tiab])	15,323
	3	1 OR 2	57,263
	4	(“HIV”[tiab] OR “Acquired Immunodeficiency Syndrome”[Mesh])	384,550
	5	3 AND 4	308
	6	((english[Filter])) NOT ((“animals”[MeSH Terms] NOT “humans”[MeSH Terms]))	25,025,079
	7	5 AND 6	256
**Embase** Host: Elsevier Data Parameters: 1947 to Present Date Searched: Date: 07/12/2022 & 11/09/2022 Searcher: Becca Billings	1	'facial affect perception':ti,ab OR 'facial affect recognition':ti,ab OR 'prosody'/exp OR 'prosody':ti,ab OR 'affect perception':ti,ab OR 'affect recognition':ti,ab OR 'emotion regulation':ti,ab OR 'theory of mind'/exp OR 'theory of mind':ti,ab OR 'mental state attribution':ti,ab OR 'affect sharing':ti,ab OR 'empathy'/exp OR 'empathy':ti,ab OR 'affect empathy':ti,ab OR 'cognitive empathy'/exp OR 'cognitive empathy':ti,ab OR 'self-other processing':ti,ab OR 'self referential processing'/exp OR 'self-referential processing':ti,ab OR 'attributional bias'/exp OR 'attribution bias':ti,ab OR 'mirror neuron'/exp OR 'mirror neuron':ti,ab OR 'social cognition'/exp OR 'social cognition':ti,ab OR 'emotion processing'/exp OR 'emotion processing':ti,ab OR 'facial emotion*':ti,ab	74,420
	2	emotion*:ti,ab AND cognit*:ti,ab AND process*:ti,ab	20,256
	3	1 OR 2	89,477
	4	hiv:ti,ab OR 'human immunodeficiency virus'/exp OR aids:ti,ab OR 'acquired immunodeficiency syndrome'/exp	607,384
	5	3 AND 4	784
	6	*Filters applied: Human, Articles*	517
**Scopus** Host: Elsevier Data Parameters: 1960 to Present Date Searched: 07/12/2022 & 11/09/2022 Searcher: Becca Billings	1	TITLE-ABS ( {facial affect perception} OR {facial affect recognition} OR prosody OR {affect perception} OR {affect recognition} OR {emotion regulation} OR {theory of mind} OR {mental state attribution} OR {affect sharing} OR empathy OR {affect empathy} OR {cognitive empathy} OR {self-other processing} OR {self-referential processing} OR {attribution bias} OR {mirror neuron} OR {social cognition} OR {emotion processing} OR {facial emotion})	86,031
	2	TITLE ( emotion* AND cognit* AND process*)	506
	3	1 OR 2	86,419
	4	TITLE-ABS ( ( *hiv* OR *{human immunodeficiency virus}* OR *aids* OR *{Acquired Immunodeficiency Syndrome}*))	567,638
	5	3 AND 4	495
	6	( LIMIT-TO ( DOCTYPE, “ar”) OR LIMIT-TO ( DOCTYPE, “re”)) AND ( LIMIT-TO ( SRCTYPE, “j”)) AND ( LIMIT-TO ( EXACTKEYWORD, “Adult”))	–
	7	*Filters applied: Article; Human*	258
		TITLE-ABS ( {facial affect perception} OR {facial affect recognition} OR prosody OR {affect perception} OR {affect recognition} OR {emotion regulation} OR {theory of mind} OR {mental state attribution} OR {affect sharing} OR empathy OR {affect empathy} OR {cognitive empathy} OR {self-other processing} OR {self-referential processing} OR {attribution bias} OR {mirror neuron} OR {social cognition} OR {emotion processing} OR {facial emotion})	86,031
		TITLE ( emotion* AND cognit* AND process*)	506

**Table 2:** Summary of HIV-related social cognitive studies (*N* = 14)

**Study**	**Participants**	**Social cognition**	**Design/protocol**	**Major findings**	**Strengths/limitations**
**Social cognitive domain—Facial emotion recognition/perception studies**					
**1. Baldonero et al. (**2013**)** Evaluation of Emotion Processing in HIV-infected Patients and Correlation with Cognitive Performance	Adults: *N* = 69 • PWH Group: *n* = 49 (*M*_*age*_ = 49 yrs) • Healthy Control Group: *n* = 20 (*M*_*age*_ = 48.5 yrs) Entry Criteria • Age 18 + • No known past or active CNS opportunistic infection or other major neurological issues (e.g., alcoholism)	• Facial Emotion Recognition Test using Ekman and Friesen photographs of 10 people (4 male & 6 female) assessing 6 basic emotions (i.e., anger, fear, disgust, happiness, sadness, surprise) (no time limit)	• 2-group cross-sectional design Primary Outcome • Associations between emotional face recognition/perception and HIV status, neurocognitive function via a performance-based battery, and HIV clinical data	• Compared to those without HIV, PWH performed worse on recognizing fear, even after controlling for age and education (*η*^*2*^ = 0.208) • Comparing PWH with (28.6%) and without (65.3%) cognitive impairment, no group differences emerged between them on fear recognition • Poorer verbal recall/memory was related to poor fear recognition (*r* = 0.42, *p* < 0.004) • Poorer overall cognitive ability (*β* = − 0.24, *p* = 0.0008) and more AIDS-defining conditions (*β* = − 0.75, *p* = 0.035) was related to poorer recognition of happiness • Facial Affect Recognition/Facial Affect Perception (effect size = − 0.306)^a^	Strengths • Control group • Validated emotional face recognition and perception test • Non-social cognition was assessed Limitations • Small *N* (< 100) • Limited to only 1 social cognition domain • Limited to cross-sectional data
**2. Clark et al. (**2010**)** Facial Emotion Recognition Impairments in Individuals with HIV	Adults: *N* = 100 • PWH Group: *n* = 50 (*M*_*age*_ = 46.2 yrs) • Healthy Control Group: *n* = 50 (*M*_*age*_ = 44.3 yrs) Entry Criteria • Native English speakers • MMSE ≥ 23 • Corrected vision • No known past or active CNS opportunistic infection or other major neurological issues (e.g., alcoholism)	• Facial Emotion Recognition Test using 84 Ekman and Friesen photographs of 12 people (6 male & 6 female) assessing 6 basic emotions (i.e., anger, fear, disgust, happiness, sadness, surprise) (no time limit)	• 2-group cross-sectional design Primary Outcome • Associations between emotional face recognition/perception and HIV status, Landscape Categorization Control Task, Benton Test of Facial Recognition, Inventory of Interpersonal Problems, Weschler Test of Adult Reading (WTAR), and HIV clinical data	• No groups differences on the ability to recognize faces; however, PWH performed significantly worse overall on recognizing facial emotions (*r* = 0.20) • PWH were less accurate to recognize fear (*t* = 2.29, *p* < 0.05) • PWH reported significantly more overall distress on interpersonal interactions (*F* = 4.23, *p* < 0.05) • Poorer facial recognition of anger was significantly associated with more distress in maintaining a sense of social connectedness (*r* = − 0.41) • Poorer overall intelligence accounted for 23% of the variance in anger recognition accuracy • Facial Affect Recognition (effect size = 0.455)^a^	Strengths • Control group • Validated emotional face recognition and perception test • Large *N* (+ 100) • Non-social cognition was assessed Limitations • Limited to only 1 social cognition domain • Limited to cross-sectional data
**3. Clark et al. (**2015**)** Facial Emotion Recognition Impairments Are Associated with Brain Volume Abnormalities in Individuals with HIV	Adults: *N* = 88 • PWH Group: *n* = 44 (*M*_*age*_ = 46.4 yrs) • Healthy Control Group: *n* = 44 (*M*_*age*_ = 44.3 yrs) Entry Criteria • Native English speakers • MMSE ≥ 23 • Corrected vision • No developmental /learning disability, or other major neurological issues (e.g., alcoholism)	• Facial Emotion Recognition Test using 84 Ekman and Friesen photographs of 12 people (6 male & 6 female) assessing 6 basic emotions (i.e., anger, fear, disgust, happiness, sadness, surprise) (no time limit)	• 2-group cross-sectional design • MRI study Primary Outcome • Associations between emotional face recognition/perception and HIV status, Landscape Categorization Control Task, Benton Test of Facial Recognition, HIV clinical data, and brain volumes	• PWH performed worse in recognizing fearful expression (*t* = 2.80, *p* = 0.006), even after controlling for education, depression symptoms, and prior opiate and cocaine use (*F* = 5.27, *p* = 0.02) • PWH had bigger amygdala volumes (*t* = 2.02, *p* = 0.05) and smaller anterior cingulate cortex volumes (*t* = 2.13, *p* = 0.04) • In PWH, recognizing fearful expression was associated with reduced anterior cingulate cortex volumes (*β* = 0.35, *p* = 0.03) • Group differences in recognizing other emotion-laden facial expressions were not detected	Strengths • Control group • Validated emotional face recognition and perception test Limitations • Small *N* (< 100) • Limited to only 1 social cognition domain • Non-social cognition was not assessed
**4. González-Baeza et al.** (2016) Facial Emotion Processing in Aviremic HIV-infected Adults	Adults: *N* = 147 • PWH Group: *n* = 107 (*M*_*age*_ = 47.4 yrs) • Healthy Control Group: *n* = 40 (*M*_*age*_ = 42.5 yrs) Entry Criteria • On ART & 2 yrs virally suppressed • No neurological comorbidities (e.g., alcoholism), active HCV treatment, vision problems, or psychiatric medications that could impair cognition	• Florida Affect Battery measures: 1) facial identity discrimination, 2) facial affect discrimination, 3) recall of the model’s face, 4) recall of the model’s facial expression, 5) facial affect naming, and 6) facial affect selection	• 2-group cross-sectional design Primary Outcome • Associations between emotional face recognition/perception and HIV status, cognition, and HIV clinical data	• PWH performed worse on recognizing the facial emotion of sadness (*z* = − 2.43, *p* = 0.015) • PWH performed worse on the facial affect selection task including sadness (*z* = − 2.52, *p* = 0.012), anger (*z* = − 2.09, *p* = 0.036), and fear (*z* = − 2.30, *p* = 0.021) • In the HIV + group with and without cognitive impairment, no group differences emerged in facial affect naming • Facial Affect Recognition/Facial Affect Naming (effect size = 0.24)^a^ • Facial Affect Recognition/Facial Affect Selection (effect size = 0.05)^a^ • Facial Affect Recognition/Facial Discrimination (effect size = 0.02)^a^ • Facial Affect Recognition/Facial Affect Discrimination (effect size = 0.22) ^a^	Strengths • Validated emotional face recognition and perception test • Control group were relatives of HIV + group & matched for age/education • Large *N* (+ 100) • Non-social cognition was assessed Limitations • Limited to only 1 social cognition domain • Limited to cross-sectional data
**5. Grabyan et al. (**2018) Deficient Emotion Processing Is Associated with Everyday Functioning Capacity in HIV-Associated Neurocognitive Disorder	Adults: *N* = 121 • PWH Group w/o HAND: *n* = 37 (*M*_*age*_ = 44.3 yrs) • PWH Group w/ HAND: *n* = 46 (*M*_*age*_ = 46.59 yrs) • Healthy Control Group: *n* = 38 (*M*_*age*_ = 43.95 yrs) Entry Criteria • No neurological comorbidities, current alcohol use, drug use, or substance use disorder (≤ 30 days)	• CogState Social Emotional Cognition Task (SECT) includes 48 trials. In each trial, 4 sets of eyes or computerized faces that convey different emotions or emotional intensities are shown at once; participants select the one that is different. The program automatically scores response speed and accuracy (age norms applied)	• 3-group cross-sectional design Primary Outcome • Associations between emotional face recognition/perception (i.e., accuracy and speed) and HIV status, cognitive status, and social aspects of everyday functioning and financial capacity as measured by the UCSD Performance-based Skills Assessment-Brief (UPSA-B)	• Compared to the HIV + group w/o HAND, those with HAND were 10 × more likely to be impaired on emotional processing accuracy • In the HAND group, the memory domain was significantly correlated to SECT accuracy (*r* = 0.4, *p* < 0.05) • The HAND group was significantly slower than the HIV + group without HAND and the healthy controls (*η*^*2*^ = 0.11, *p* < 0.001) • In PWH, accuracy independently predicted functional capacity tapping social ability (*F* = 5.4, adjusted *R*^*2*^ = 0.1, *p* = 0.007) but not for financial capacity; however, this was not found for reaction time • Facial Affect Recognition/SECT (effect size = 0.330)^a^	Strengths • Validated emotional face recognition and perception test with age norms • Control group • Large *N* (+ 100) • Non-social cognition was assessed Limitations • Limited to only 1 social cognition domain • Limited to cross-sectional data
**6. Heilman et al. (**2013**)** Atypical Autonomic Regulation, Auditory Processing, and Affect Recognition in Women with HIV	Women: *N* = 83 • Women with HIV Group: *n* = 61 (*M*_*age*_ = 42.16 yrs) • Healthy Women Group: *n* = 22 (*M*_*age*_ = 38.09 yrs) Entry Criteria • No illicit drug use within 24 h, prior psychiatric history, uncontrolled medical condition, current HCV viremia, visual or auditory deficit, heart condition, stroke, or current pregnancy, postpartum, or lactating	• Dynamic Affect Recognition Evaluation (DARE) measures facial emotion recognition. It entails the presentation of neutral faces that gradually morph to 1 of 6 targeted emotional expressions (i.e., happiness, anger). During these trials, participants indicate as soon as they recognize the emotion (latency) and verbally identify it (accuracy)	• 2-group cross-sectional design Primary Outcome • Associations between affective recognition, auditory processing (i.e., filtered words vs competing words), and heart rate variability (HRV) as measured by respiratory sinus arrhythmia (RSA), and non-social cognition (intelligence—WTAR) were examined to explore degrading effects of HIV within the context of the Social Engagement Theory	• RSA was lower in women with HIV (*ηρ*^*2*^ = 0.07, *p* < 0.02) • Women with HIV performed worse on the accuracy of the DARE test (*p* < 0.03), although there was no group difference on any specific emotion nor was there a difference in latency of DARE test • Across both the women with and without HIV, greater intelligence (WRAT) was related to fewer accuracy errors on DARE sad (*r* = − 0.25, *p* < 0.03), disgust (*r* = − 0.28, *p* < 0.02), anger (*r* = − 0.45, *p* < 0.000), and total videos (*r* = − 0.36, *p* < 0.002) • In women with HIV, greater intelligence was related to fewer accuracy errors on DARE across all videos (*r* = − 0.34, *p* < 0.02) and the anger videos (*r* = − 0.48, *p* < 0.000) • Facial Affect Recognition/Dynamic Affect Recognition Evaluation (effect size = 0.471)^a^	Strengths • Validated emotional face recognition and perception test • Control group • Non-social cognition was assessed • Examined Social Engagement Theory with measures of HRV and auditory processing Limitations • Small *N* (< 100) • Auditory processing task was neutral (i.e., did not contain emotional valence) • Limited to 1 social cognition domain • Limited to cross-sectional data
**7. Kamkwalala (**2021) Sex-specific Effects of Low-dose Hydrocortisone on Threat Detection in HIV	PWH: *N* = 65 • Women with HIV Group: *n* = 31 (*M*_*age*_ = 36.40 yrs) • Men with HIV Group: *n* = 34 (*M*_*age*_ = 31.85 yrs) Entry Criteria • English as 1st language • On ART ≥ 3 months • No history of substance abuse in 6 months, clean urine toxicology within 24 h, BMI > 40 kg/m^2^, neurological condition affecting cognition	• Facial Emotion Perception Test (FEPT) is a computerized task in which pictures of animals and people are shown to participants, who rate the emotional valence of each image. Accuracy and reaction time are outcomes	• 2-group, double-blind, placebo-controlled, cross-over design • A pharmacologic challenge study • Single low-dose of hydrocortisone (LDH 10 mg) Primary Outcome • Associations between emotional face recognition/perception (i.e., accuracy and speed) by elevated glucocorticoids (LDH vs placebo) and sex, non-social cognition, childhood trauma, and self-reported stress/anxiety	• Accuracy in FEPT, especially for fearful faces, was influenced by LDH in women only (*d* = 0.44, *p* = 0.04) • This threat detection (i.e., sensitivity to detect fearful faces) was more definite in those women with lower overall childhood trauma (*r* = − 0.40, *p* = 0.02), lower childhood emotional abuse (*r* = − 0.35, *p* = 0.05), lower childhood physical neglect (*r* = − 0.43, *p* = 0.01), lower childhood physical abuse (*r* = − 0.49, *p* = 0.005), and lower anxiety (*r* = − 0.32, *p* = 0.08) • Relationship to accuracy of identifying fearful faces with neurocognitive measures were mixed	Strengths • Sex differences examined • Unique experimental design with LDH • Validated emotional face recognition and perception test • Non-social cognition was assessed Limitations • Small *N* (< 100) • Limited to only 1 social cognition domain • Limited to cross-sectional data
**8. Lane et al. (**2012**)** Facial Emotional Processing in HIV Infection: Relation to Neurocognitive and Neuropsychiatric Status	Adults: *N* = 110 • PWH Group: *n* = 85 (*M*_*age*_ = 55.40 yrs) • Healthy Control Group: *n* = 25 (*M*_*age*_ = 53.68 yrs) Entry Criteria • 45 yrs or older • On ART stably for + 6 months • Nadir CD4 ≤ 350 • HIV dx + 5 yrs • English proficiency • No neurological or psychiatric disorder, current alcohol or substance use disorders, history of syncope, heavy marijuana use, and active HCV	• The University of Pennsylvania Computerized Neuropsychological Test Battery Version 2, Face and Emotion Tasks in which 3 subtests were used • Penn Emotion Recognition Task (i.e., identify the emotion of a face) • Penn Emotion Discrimination Task (i.e., decide whether two faces express equivalent emotional intensity) • Penn Emotional Acuity Test (i.e., determine emotional value of a face)	• 2-group cross-sectional design Primary Outcome • Associations between emotional face recognition/perception and HIV status, cognitive status, depressive symptomatology, apathy, everyday functioning (i.e., self-reported IADLS), and HIV clinical data	• PWH were slower in recognizing fearful faces (*d* = 0.37, *p* = 0.04), poorer in discriminating intensity of happy expressions (*d* = 0.52, *p* = 0.02), and identifying sad expressions (*d* = 0.43, *p* = 0.02) • PWH with HAND had: 1) slower recognition of happiness (*d* = 0.63, *p* < 0.03), anger (*d* = 0.69, *p* < 0.03), and sadness (*d* = 0.88, *p* < 0.007), and 2) poorer discrimination of happiness (*d* = 0.83, *p* < 0.003) • In PWH, poorer overall neurocognitive performance was associated with poorer discrimination of happiness (*R*^*2*^ = 0.22, *p* < 0.001) • In PWH, slower reaction for recognizing fearful faces was associated with poorer mental flexibility (*R*^*2*^ = 0.076, *p* = 0.01), speed of information processing (*R*^*2*^ = 0.076, *p* = 0.01), and motor function (*R*^*2*^ = 0.054, *p* = 0.04) • In PWH, poorer discrimination of happy faces was associated with poorer speed of information processing (*R*^*2*^ = 0.17, *p* < 0.0001), motor function (*R*^*2*^ = 0.08, *p* < 0.009), and mental flexibility (*R*^*2*^ = 0.11, *p* < 0.002) • Facial Affect Recognition/Penn Emotion Recognition (effect size = 0.314)^a^	Strengths • Validated emotional face recognition and perception test, with several varieties of measuring emotional facial perception • Control group • Good *N* (+ 100) • Non-social cognition was assessed Limitations • Limited to only 1 social cognition domain; but had multiple tests of emotional faces • Limited to cross-sectional data
**9. Lysaker et al. (**2014**)** Deficits in Metacognitive Capacity Distinguish Patients with Schizophrenia from Those with Prolonged Medical Adversity	Adults: *N* = 217 • Schizophrenia Group: *n* = 166 (*M*_*age*_ = 48.4 yrs) • PWH (control) Group: *n* = 51 (*M*_*age*_ = 48.3 yrs) Entry Criteria • No mental retardation or active substance use • Participants with schizophrenia could not have HIV or hospitalization in past 30 days • PWH (no history of psychosis)	• Bell-Lysaker Emotional Recognition Task (BLERT) uses videotape of actors for participants to identify/rate emotional affect	• 2-group cross-sectional design with the PWH group serving as the comparative/control group who has medical adversity Primary Outcome • Comparison between adults with schizophrenia and PWH on the BLERT	• The schizophrenia group performed slightly worse (*M* = 12.8; *SD* = 0.3) than the HIV group (*M* = 14.1, *SD* = 0.4), but both groups were not vastly different on the BLERT. This suggests that PWH may be functioning at a similar level with those with schizophrenia who have difficulty recognizing or processing facial affect	Strengths • BLERT is a well- established measure of social cognition • Large *N* (+ 100) Limitations • Non-social cognition was not assessed • No healthy control group to compare the HIV + group • Limited to only 1 social cognition domain • Limited to cross-sectional data
**10. Rubin et al. (**2022a**, **b**)** Early Life Trauma and Social Processing in HIV: The Role of Neuroendocrine Factors and Inflammation	PWH: *N* = 58 • PWH with Early Life Trauma (ELT): *n* = 29 (*M*_*age*_ = 32.7 yrs) • PWH without ELT: *n* = 29 (*M*_*age*_ = 34.6 yrs) Entry Criteria • On ART ≥ 3 months • English as 1st language • No history of substance abuse in 6 months, clean urine toxicology within 24 h, BMI > 40 kg/m^2^, neurological condition affecting cognition	• Facial Emotion Perception Test (FEPT) is a computerized task in which pictures of animals and people are shown to participants, who rate the emotional valence of each image. Accuracy and reaction time are outcomes	• 2-group cross-sectional study of secondary data analysis of a placebo group in a pharmacologic challenge study Primary Outcome • Comparison of FEPT between PWH with and without ELT (i.e., Childhood Trauma Questionnaire) • Examine whether these neurobiological factors (cytokines, oxytocin, arginine vasopressin) impact FEPT performance across groups	• Those with ELT performed worse on accuracy of FEPT (*p* = 0.021, *d* = 0.63) • Those with ELT also had higher levels of oxytocin (*p* = 0.039, *d* = 0.57) but not arginine vasopressin • Those with ELT with low (oxytocin & CRP) performed worse on the accuracy of the FEPT (*p* < 0.05) • Those who had higher levels of myeloid migration, regardless of ELT, performed worse on total recognition accuracy (β = − 0.39; *p* = 0.0026) as well as for happy (β = − 0.33; *p* = 0.017), angry (β = -0.32; *p* = 0.018), sad (β = − 0.32; *p* = 0.022), fearful (β = − 0.28; *p* = 0.038), and neutral faces (β = − 0.24; *p* = 0.061) • In those with ELT, a trend emerged whereby those higher on HPA axis hormones performed worse on total recognition accuracy (*p* = 0.099 for interaction) than those without early life trauma	Strengths • Validated emotional face recognition and perception test • Investigated neurobiological mechanisms that regulate social cognition Limitations • Small *N* (< 100) • Limited to 1 social cognition domain • Non-social cognition was not assessed • Limited to cross-sectional data
**Social Cognitive Domain – Prosody Study**					
**11. González-Baeza et al.** (2017) Vocal Emotion Processing Deficits in HIV-infected Individuals	Adults: *N* = 146 • PWH Group: *n* = 100 (*M*_*age*_ = 47.5 yrs) • Healthy Control Group: *n* = 46 (*M*_*age*_ = 45.5 yrs) Entry Criteria • On ART for 2 yrs & virally suppressed • No neurological comorbidities (e.g., alcoholism), active HCV treatment, vision problems, or psychiatric medications that could impair cognition	• Prosody Test – Based on the Florida Affect Battery 9th subtest, this test required participants to listen to 9 different auditory sentences that were emotionally-ladened (i.e., fear, happiness, sadness, anger, neutral). They matched these sentences to faces expressing the same emotion	• 2-group cross-sectional design • MRI & MRS (subset *n* = 36 HIV +) Primary Outcome • Associations between prosody and HIV status, non-social cognition, HIV clinical data, and brain volumes	• PWH performed worse on the vocal prosody test (*t* = 2.07, *p* = 0.04) • Those PWH without HAND performed similarly on the vocal prosody test (*t* = 1.09, *p* = 0.28) compared to the healthy controls • PWH with HAND performed worse on the vocal prosody test (*t* = 3.19, *p* = 0.002) compared to healthy controls • Controlling for non-social cognition, positive associations between vocal prosody and brain volumes were detected in left hippocampus (*RC* = 0.0010056, *p* = 0.029), right temporal (*RC* = 0.000073, *p* = 0.03) and parietal lobes (*RC* = 0.0000918, *p* = 0.05), left (*RC* = 0.009576, *p* = 0.002) and right thalamus (*RC* = 0.000979, *p* = 0.002) • Prosody/Prosody Perception (effect size = 0.399)^a^	Strengths • Control group included relatives of HIV + group & matched for age, education, and gender • Large *N* (+ 100) • Non-social cognition was assessed • Connects prosody along with emotional face recognition; taps into 2 social cognitive domains Limitations • Limited to cross-sectional data
**Social cognitive domain—Theory of mind studies**					
**12. Homer et al. (**2013**)** Methamphetamine Use and HIV in Relation to Social Cognition	Adults: *N* = 57 (*M*_*age*_ = 40 yrs) • Meth User plus + HIV Group: *n* = 13 • Meth User plus -HIV Group: *n* = 16 • Non-Meth User plus + HIV Group: *n* = 19 • Non-Meth User plus -HIV Group: *n* = 8 Entry Criteria • English speaking • No abuse or dependence on any other drugs • Identify as a man who has sex with men • Non-Meth users could not have a history of meth use	• Faux Pas Recognition Task consists of 20 brief stories, half with a faux pas. 1 point is awarded for each story in which participants identify whether a faux pas occurred or not; providing a correct rationale for why something was socially awkward results in another point • Reading the Mind in the Eyes Task entails looking at 36 faces and use a word list to describe the emotionality in each face	• 4-group, cross-sectional study Primary Outcome • Associations between meth use and HIV status on measures of facial emotion and theory of mind	• Meth users performed worse on the Eyes Task than nonusers (*p* = 0.004, partial *η*^*2*^ = 0.15) • PWH performed worse on the Eyes Task than those without HIV (*p* = 0.02, partial *η*^*2*^ = 0.10) • The interaction between meth use and HIV status on the Eyes Task was not significant (*p* = 0.38, *d* = 0.24) • In the Faux Pas Task, there was a significant main effect in that meth users performed worse (*p* = 0.03, partial *η*^*2*^ = 0.08) • Although PWH performed worse on the Faux Pas Task than those without HIV, this difference was not significant (*p* = 0.28, *d* = − 0.45) • The interaction between meth use and HIV status on the Faux Pas Task was not significant (*p* = 0.71, partial *η*^*2*^ = 0.00) • Theory of Mind/Eyes Task (effect size = 0.829)^a^ • Theory of Mind/Faux Pas Task (effect size = 0.438)^a^	Strengths • Control groups for meth use and HIV status • Acceptable measures of social cognition • Assesses both theory of mind and emotional face recognition • Executive function was measured Limitations • Small *N* (< 100) • Non-social cognition was not fully assessed • The amount of meth use was not incorporated • Limited to cross-sectional data
**13. Lysaker et al. (**2012**)** Metacognitive and Social Cognition Deficits in Patients with Significant Psychiatric Adversity	Adults: *N* = 65 • Schizophrenia Group: *n* = 40 (*M*_*age*_ = 48.84 yrs) • PWH (control) Group: *n* = 25 (*M*_*age*_ = 51.68 yrs) Entry Criteria • No mental retardation or active substance use • Participants with schizophrenia could not have HIV or hospitalization in past 30 days • PWH (no history of psychosis)	• Hinting Test assesses theory of mind by having participants make judgement about intentions of fictional characters • Metacognition Assessment Scale (MAS) assessed metacognitive abilities centering on formation and integration of self and others • Bell-Lysaker Emotional Recognition Task (BLERT) uses videotape of actors for participants to identify/rate emotional affect	• 2-group cross-sectional design with HIV + group serving as the comparative (control) group who has medical adversity Primary Outcome • Associations between emotional face recognition and perception, theory of mind, metacognition, and verbal learning and memory (i.e., Hopkins Verbal Learning Test)	• The schizophrenia group performed significantly worse on the Hinting Test (*F* = 5.97, *p* = 0.02) and all the MAS subscales (Self-reflectivity, *F* = 12.62, *p* = 0.001; Understanding of Other’s Mind, *F* = 20.42, *p* > 0.001; Decentration, *F* = 8.19, *p* = 0.006; Master, *F* = 16.54, *p* > 0.001). This suggests that PWH have better theory of mind and metacognition ability than those with schizophrenia • Both groups were not different on the BLERT (*F* = 0.27); this suggests that PWH may be functioning at a similar level with those with schizophrenia who have difficulty recognizing or processing facial affect	Strengths • Validated emotional face recognition perception test • Integrated other measures of social cognition (i.e., theory of mind, facial emotional recognition) • HIV + control group Limitations • Small *N* (< 100) • Non-social cognition was not fully assessed, only verbal learning and memory • Limited to cross-sectional data
**Social cognitive domain—Empathy study**					
**14. Walzer et al. (**2016**)** Other- Versus Self-focus and Risky Health Behavior: The Case of HIV/AIDS	Adults: *N* = 79 • PWH (*M*_*age*_ = 41.86 yrs) Entry Criteria • 21 years old or older • Being HIV +	• Other Perspective-Taking measured with a 7-item scale whereby one indicates the degree one takes on the perspective of others • Empathic Concern measured with a 7-item scale in which one indicates to what degree he/she empathizes with others	• 1-group cross-sectional design Primary Outcome • Associations between empathic concern and other perspective taking (i.e., theory of mind) on one’s willingness to engage in 11 HIV/AIDS risk behaviors (i.e., “sharing a needle while shooting up drugs with friends”)	• Those with more other-focused perspectives indicated that they were less likely to engage in risky behaviors while a self-focused perspective was associated with greater willingness to engage in such risk behaviors	Strengths • Unique focus on empathy and theory of mind on willingness to engage on HIV/AIDS risk behaviors Limitations • Small *N* (< 100) • No control group • Non-social cognition was not assessed • No inclusion of other social cognition measures • Limited to cross-sectional data

*BLERT* Bell-Lysaker emotional recognition, *CNS* central nervous system, *CRP* C-reactive protein, *DARE* dynamic affect recognition evaluation,* ELT* early life trauma, *FEPT* facial emotion perception test, *HAND *HIV-associated neurocognitive disorder, *HCV* hepatitis C, *HPA* hypothalamus–pituitary–adrenal (axis), *HRV* heart rate variability,* IADL* instrumental activities of daily living, *LDH* low-dose hydrocortisone, *MMSE* mini mental state examination, *MoCA* Montreal cognitive assessment, *MRI* magnetic resonance imaging,* MRS* magnetic resonance scan, *RSA* respiratory sinus arrhythmia, *SECT* social emotion cognition task, *UCSD* university of California San Diego, *UPSA-B* UCSD performance-based skills assessment-brief, weeks wks, *WTAR* Weschler test of adults reading, *years *yrs

^a^reported effects sizes used in meta-analysis

**Table 3:** Rigor of prior social cognition studies in PWH

**Study**	**Social Cognitive Domains**				**Sample size ***N*** > 100**	**Control group/comparison group**	**Study design**	**Non-social cognition measures**	**Neuro-logical measures**	**Social everyday function measures**	**Stigma**
	**Facial emotion recognition/perception**	**Prosody**	**Theory of mind**	**Empathy**							
Baldonero et al. (2013)	**FERT**	no	no	no	no *N* = 69	HIV-neg	Cross-sectional	**YES** Full Battery	no	No	no
Clark et al. (2010)	**FERT**	no	no	no	**YES** *N* = 100	HIV-neg	Cross-sectional	**YES** WTAR	no	**YES**	no
Clark et al. (2015)	**FERT**	no	no	no	no *N* = 88	HIV-neg	Cross-sectional	No	**MRI**	No	no
González-Baeza et al. (2016)	**FAB**	no	no	no	**YES** *N* = 147	HIV-neg	Cross-sectional	**YES** Full Battery	no	No	no
González-Baeza et al. (2017)	no	**FAB**	no	no	**YES** *N* = 146	HIV-neg	Cross-sectional	**YES** Full Battery	**MRI & MRS**	No	no
Grabyan et al. (2018)	**SECT**	no	no	no	**YES** *N* = 121	HIV-neg	Cross-sectional	**YES** HAND	no	**YES**	no
Heilman et al. (2013)	**DARE**	no	no	no	no *N* = 83	HIV-neg	Cross-sectional	**YES** WTAR	**HRV & RSA**	No	no
Homer et al. (2013)	**RMET**	no	**FPRT**	no	no *N* = 56	HIV-neg	Cross-sectional	**YES** Stroop	no	No	no
Kamkwalala et al. (2021)	**FEPT**	no	no	no	no *N* = 65	HIV + men HIV + women	Cross-over	**YES** Full Battery	**Drug challenge**	No	no
Lane et al. (2012)	**UPFET**	no	no	no	**YES** *N* = 110	HIV-neg	Cross-sectional	**YES** Full Battery	no	No	no
Lysaker et al. (2012)	**BLERT**	no	**Hinting Test**	no	no *N* = 65	schizophrenia group	Cross-sectional	**YES** HVLT	no	no	no
Lysaker et al. (2014)	**BLERT**	no	no	no	**YES** *N* = 217	schizophrenia group	Cross-sectional	no	no	no	no
Rubin et al., (2022a, b)	**FEPT**	no	no	no	no *N* = 58	PWH w/ ELT PWH w/o ELT	Cross-sectional	no	**Neuro-markers**	no	no
Walzer et al. (2016)	no	no	no	**7-item scales**	no *N* = 79	no	Cross-sectional	no	no	no	no

There were no longitudinal or intervention studies

*BLERT* Bell-Lysaker Emotional Recognition Task, *DARE* dynamic affect recognition evaluation, *ELT* early life trauma, *FAB* Florida Affect Battery, *FEPT* facial emotion perception test, *FERT* facial emotion recognition test, *FPRT* Faux pas recognition task, *HAND* HIV-associated neurocognitive disorder, *HRV* heart rate variability,* HVLT* Hopkins verbal learning test, *MRI* magnetic resonance imaging, *MRS* magnetic resonance scans, *PWH* people with HIV, *RMET* reading the mind in the eyes task, *RSA* respiratory sinus arrhythmia, *SECT* CogState social emotional cognition task, *UPFET* University of Pennsylvania Face and Emotion Tasks, *WTAR *Weschler test of adult reading

### Summaries

Of the studies (*n* = 14) that met the criteria, most examined facial emotion recognition/perception, followed by two theory of mind studies, one prosody (vocal emotion processing) study, and one empathy study; three of these studies examined more than one type of social cognition. Yet, many of these studies also examined several variables that predicted the performance in these social cognition constructs including non-social cognition, volume of brain regions, EEG, biological sex, and early life trauma. The articles are summarized in alphabetical order by the article’s first author in Appendix as well as in Table 2 grouped by type of social cognitive domain.

### Systematic Review Synthesis of Findings

Based on the systematic review, from these studies, converging evidence suggests that some PLHIV may experience mild to moderate deficits in social cognition, which are influenced by early life trauma, biological sex, and sometimes non-social cognitive functioning. Regarding facial emotion recognition/perception, compared to a control group (without HIV), PLHIV were found to be less accurate and/or slower in identifying emotional valence of faces in seven studies (Baldonero et al., 2013; Clark et al., 2010, 2015; González-Baeza et al., 2017; Grabyan et al., 2018; Heilman et al., 2013; Homer et al., 2013; Lane et al., 2012). Difficulty with facial emotion recognition/perception was observed across the spectrum of basic emotions (i.e., anger, fear, disgust, happiness, sadness, surprise); however, more difficulty with recognizing/perceiving faces with negative emotional content was particularly noted in several of these studies (Baldonero et al., 2013; Clark et al., 2010, 2015; González-Baeza et al., 2017; Lane et al., 2012). In two studies in which PLHIV served as a control group with medical adversity (Lysaker et al., 2012, 2014), those with schizophrenia performed worse on facial emotion recognition/perception tasks, but they were not statistically different; this suggests that some PLHIV may be functioning at a similar level as those with schizophrenia who have well documented deficits in social cognition (Green et al., 2015, 2019; Mier & Kirsch, 2017).

Focusing further on facial emotion recognition/perception, predictors of poor performance of PLHIV included early life trauma, neurobiological factors, and non-social cognition. Both Kamkwalala et al. (2021) and Rubin et al. (2022a, b) observed that those with early life trauma experienced more difficulty in facial emotion recognition/perception tasks. The findings from the Kamkwalala et al. (2021) study suggest that this pattern may be more prominent for fearful faces in women living with HIV who did not report early life trauma. As a pharmacologic challenge with placebo vs low dose hydrocortisone, Kamkwalala et al. (2021) also found that low dose hydrocortisone enhanced threat detection of fearful faces, but only in women; within women, the effect was more prominent in women with severe early life trauma. This suggests that the HPA axis plays a role in fearful face recognition in women (specifically with severe early life trauma), but this hydrocortisone effect was blunted in general for men. Further, Rubin et al. (2022a, b) observed those with early life trauma had higher oxytocin levels; and those with early life trauma with low oxytocin and C-reactive protein levels performed worse on the Facial Emotion Perception Test. Further, those with higher levels of myeloid migrations (i.e., MCP-1, MMP-9), regardless of early life trauma, performed worse on total recognition accuracy including happy, angry, sad, fearful, and neutral faces. These findings suggest that those with higher HPA axis hormones performed worse on total facial emotion recognition accuracy than those without early life trauma who have more normal oxytocin levels.

Brain circuitry and non-social cognition undoubtedly influence the social cognition of facial emotion recognition/perception to some degree. Clark et al. (2015) observed that compared to those without HIV, PLHIV had larger amygdala volumes and smaller anterior cingulate cortex volumes; furthermore, recognizing fearful expressions was associated with reduced anterior cingulate cortex volumes. Coupled with the HPA connections as described by Kamkwalala et al. (2021) and Rubin et al. (2022a, b), this suggests neurological involvement with social cognitive impairments, consistent with the potential effect of HIV pathogenesis on brain health (Waldrop et al., 2021). Several studies also examined the effect of non-social cognition on social cognition in PLHIV. Nine studies on the facial emotion recognition/perception included measures of non-social cognition. Four out of nine studies found that poorer non-social cognition was related to poorer facial emotion recognition/perception (Clark et al., 2010; Grabyan et al., 2018; Heilman et al., 2013; Lane et al., 2012); one found no association (González-Baeza et al., 2016); and two found mixed results (Baldonero et al., 2013; Kamkwalala et al., 2021). For example, Baldonero et al. (2013) compared PLHIV with (28.6%) and without (65.3%) non-social cognitive impairment and found no group differences on fear recognition, but they did find that poorer verbal recall/memory was related to poorer fear recognition and overall non-social cognitive ability was related to poorer recognition of happiness. With these findings, the evidence converges that non-social cognition likely plays a role in facial emotion recognition/perception. However, as non-social cognition was not consistently assessed across studies, and the major non-social cognitive domains (e.g., verbal memory, executive functioning) were not always represented in the non-social cognitive batteries, it remains unclear whether social cognitive impairments of PLHIV are entirely explained by non-social cognitive impairments. This overall pattern of findings mirrors the larger social cognitive literature (Rubin et al., 2022a, b).

Prosody perception was represented in only one study. Using a prosody test composed of nine different auditory sentences that were emotionally ladened (i.e., sadness, anger, happiness, fear, neutral), González-Baeza et al. (2017) found PLHIV performed worse on the vocal prosody test than controls. While PLHIV without HAND and controls showed comparable performance on the prosody perception task, PLHIV with HAND performed significantly worse on prosody than healthy controls. Better prosody scores were also associated with larger brain volumes in the left hippocampus, right temporal and parietal lobes, and right thalamus. This study of prosody perception suggests that deficits in prosody perception in PLHIV may be influenced by poorer non-social cognition.

Theory of mind was represented in three very different studies. Homer et al. (2013) examined both theory of mind and facial emotion recognition/perception in those with or without methamphetamine use and those living with or without HIV. Both methamphetamine use and PLHIV performed worse on the facial emotion recognition/perception test than those who do not use methamphetamine and those living without HIV, respectively. Regarding the Faux Pas Task (a theory of mind test), there was a significant main effect in that methamphetamine users performed worse than methamphetamine non-users. While PLHIV also performed worse on the Faux Pas Task than those living without HIV, this difference was not statistically significant. Further, the interaction between methamphetamine use and HIV status on the Faux Pas Task was not significant. The other theory of mind study by Lysaker et al. (2012) found that in comparison to those with schizophrenia, PLHIV performed significantly better on the theory of mind test (Hinting test) and a metacognition test (Metacognition Assessment Scale); however, on a measure of facial emotion recognition/perception (BLERT), there were no group differences. Overall, these studies suggest there may be some minor deficits in theory of mind for PLHIV, but without more studies with control groups, it is difficult to draw any firm conclusion.

Empathy was assessed in only one study. Walzer et al. (2016) observed that those with more empathy (i.e., other focused perspectives) were less likely to engage in risky behaviors that could have an impact on others. Unfortunately, this was a single group study that did not include any control comparison group and employed only a self-report questionnaire. Thus, further studies are needed to better understand whether PLHIV show impaired empathy or not.

In these 14 studies on social cognition, the impact on social everyday functioning was rarely examined. Clark et al. (2010) found that poorer facial emotion recognition/perception of anger was significantly associated with more distress in maintaining a sense of social connectedness. Grabyan et al. (2018) found in PLHIV that accuracy in facial emotion recognitive/perception was related to social ability (i.e., Communication subscale task of the UCSD Performance-based Skills Assessment-Brief). These two studies demonstrate that aspects of social cognition impact everyday social activity, as would be expected. It will be important for future studies to assess diverse aspects of social functioning, including objective social isolation (i.e., social network size, the diversity of social network), subjective social isolation (i.e., quality of social network, loneliness), and integration into a social support system, and map out the extent to which social cognition affects social functioning in PLWH.

## Meta-Analysis of Social Cognition in the Context of HIV Infection

To quantify social cognitive performance of PLWH compared to people living without HIV, we conducted a meta-analysis using a correlated effects model. Among relevant studies reviewed above (also see Table 2), eight studies were qualified for this analysis, as all of them included PLWH and people living without HIV, administered at least one social cognitive measure, and provided effect sizes on the group difference or necessary information to calculate effect sizes (e.g., the mean and standard deviation of two groups, or reported the effect size). Studies that did not include a control group of people living with HIV and did not employ a performance task on social cognition were not included in meta-analysis as this can limit how the data can be combined (i.e., data need to be gathered in similar ways for the results to be meaningful). See Table 2 for study characteristics and effects sizes of included studies in the meta-analyses. As some studies reported more than one effect size using the same sample, the single-level meta-analysis approach that assumes independence of effect sizes is not appropriate. Thus, we decided to fit a correlated effects model using the robust variance estimation (RVE) framework with an adjustment for small sample size (Hedges et al., 2010; Pustejovsky & Tipton, 2022; Tipton, 2015). The proportion of heterogeneity associated with differences between studies was evaluated with the I^2^ statistic. All analyses were performed using the R implementation of these algorithms in the robumeta package Fisher and Tipton (2015).

**Fig. 2:**
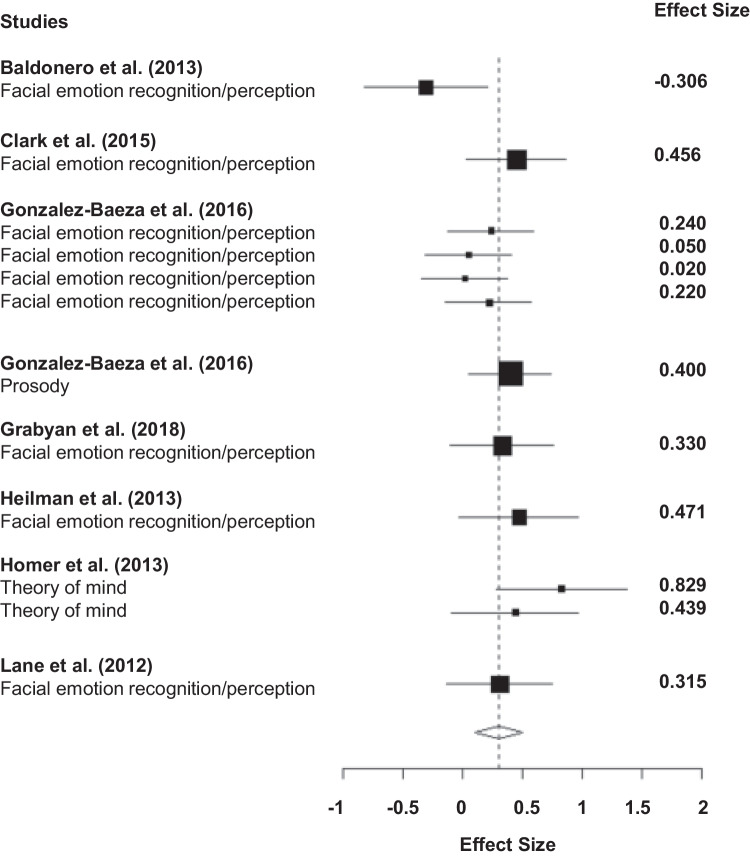
Forest plot for the difference in social cognitive performance between those with and without HIV

**Fig. 3:**
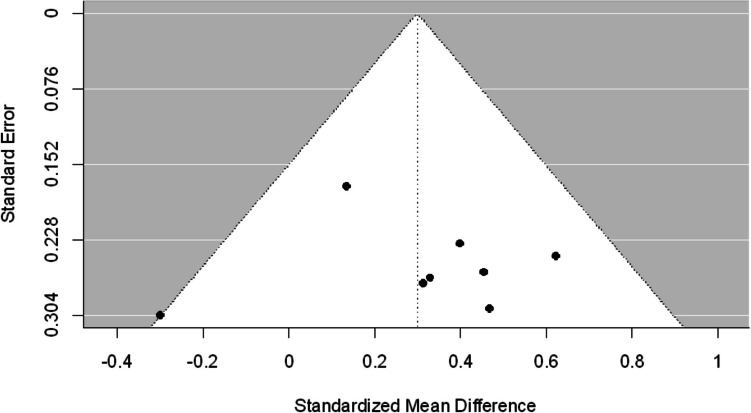
Funnel plot for across social cognition domains

For this analysis, eight studies provided 12 observed effect sizes between two groups: nine effect sizes on facial affect perception, one effect size on prosody perception, and two effect sizes on theory of mind. Specifically, Gonzelez-Baeza et al. (2016) provided four effect sizes on facial affect perception and Homer et al. (2013) provided two effect sizes on theory of mind. All the other studies provided one effect size. Across eight studies, the estimated effect size between two groups was 0.305 (95% confidence interval: 0.108, 0.502) (see Fig. 2 for details). We did not find evidence of excessive heterogeneity between studies (I^2^ = 26, T^2^ = 0.02) (see Fig. 3).

In addition, we conducted another meta-analysis on facial affect perception alone. Across the six studies reporting facial affect perception the estimated effect size was 0.24 3 (95% confidence interval: − 0.016, 0.502, I^2^ = 27, T^2^ = 0.02) (see Fig. 2). We suspect that this result is due to the outlier results of Baldonero et al. (2013) in this subset of studies. While these findings indicate that overall PLWH show impaired social cognitive performance compared to people living without HIV, this finding should be considered in the context of a small number of studies that were included in this analysis and the limited scope of included social cognitive domains.

## Discussion

Methodologically, these social cognition studies in the context of HIV infection have distinct and overlapping features that influence the quality of the data and the conclusions derived from them. Such features include the types of social cognitive domains measured, sample size, adequacy of control group(s), study design, inclusion of a non-social neuropsychological test battery, inclusion of neurological measures (e.g., biomarkers, MRI), inclusion of social everyday outcome measures (i.e., social networks, social communication), and other influential factors (i.e., substance use). To guide this methodological synthesis, Table 3 highlights the limitations of these 14 studies, thus indicating emerging growth opportunities for the social cognition HIV literature.

### Social Cognition Measures

Social cognitive ability is generally assessed through measures of facial emotion recognition/perception, prosody, theory of mind, and empathy. In our systematic review, social cognition is represented in 12 facial emotion recognition/perception studies, two theory of mind studies, one prosody study, and one empathy study, with some overlap in domains in two studies. One study on empathy employed a self-report questionnaire, rather than using a performance task. For a more complete picture of social cognition in the context of HIV infection, studies are needed that assess all the main social cognitive concepts and corresponding measures within the same study, to determine whether each social cognitive domain is similarly affected among PLHIV or not, and the extent to which each social cognitive domain is related to other domains.

### Sample Size

Samples sizes for these studies ranged from 56 to 217 (i.e., 56, 58, 65, 65, 69, 79, 83, 88, 100, 110, 121, 146, 147, 217). As such, these studies were adequately powered to satisfy the central limit theorem needed for most general statistical analyses. Half were under 100, as such there is some concern that such limited sample sizes may reduce their generalizability and ecological validity. As few studies report power analyses, none of the studies reported whether they were adequately powered or not; however, several reported effect sizes (Grabyan et al., 2018; Heilman et al., 2013; Homer et al., 2013; Kamkwalala et al., 2021; Lane et al., 2012; Rubin et al., 2022a, b). Ultimately, small sample sizes prohibit our ability to carefully examine the role of biological variables and social categories on social cognition in PLHIV (e.g., age, sex assigned at birth, gender identity, sexual identity).

### Control Group Adequacy

Most (13 out of 14) studies had some sort of control/comparison group, which provided a context to the findings. Nine of these provided a comparison with people living without HIV which provides an adequate control group to infer the effect of HIV infection on social cognitive processes. Sex differences in PLHIV were examined in one study (i.e., Kamkwalala et al., 2021); although this study did not include the HIV-negative comparison, it was laudable to compare performance between men and women as sex differences on social cognitive processes have been observed in other populations (Ferrer-Quintero et al., 2021; Proverbio, 2023). Likewise, Rubin et al. (2022a, b) compared those with and without early life trauma in PLHIV; this provided a unique comparison on an obvious important variable related to social cognition (Rokita et al., 2020). Two studies (Lysaker et al., 2012, 2014) employed PLHIV as a control group for those with schizophrenia; it is harder to interpret these findings within a larger context, but it did provide a unique comparison to a population that has well documented deficits in social cognition (Lee et al., 2013; Mier & Kirsch, 2017).

### Study Design

These studies were all cross-sectional protocols; although the Kamkwalala et al. (2021) study was a placebo-controlled cross-over study. Thus, we do not know whether social cognitive processes in PLHIV are stable or change over time. As non-social cognition in PLHIV often vacillates over time (Vance et al., 2022), social cognition may also vacillate depending on a host of factors such as how well one learns to cope with medical adversity, changes in cognition, and mood. Thus, it is important to document the durability of social cognitive functioning overtime in longitudinally designed studies.

### Non-Social Neuropsychological Assessment

Ten out of 14 studies assessed non-social cognition to some degree (six with full non-social neuropsychological test batteries). Some studies suggest a connection between social cognition and non-social cognition (Clark et al., 2010; Grabyan et al., 2018; Heilman et al., 2013; Lane et al., 2012), one found no connection (González-Baeza et al., 2016), and two found mixed results (Baldonero et al., 2018; Kamkwalala et al., 2021). Thus, social cognitive ability is related to non-social cognitive ability to some degree in PLHIV, a pattern reflective of what has been shown in other populations (Cella et al., 2015) but not all (i.e., schizophrenia, Rubin et al., 2022a, b). Future studies are needed to determine the extent to which non-social cognitive impairments explain social cognitive impairments in PLHIV. Further, none of the studies (Table 3) included measures of subjective non-social cognition such as self-rated non-social cognitive health. NeuroHIV studies vary as to whether subjective non-social cognition may or may not be related to objective non-social neuropsychological test performance (Jacob et al., 2022; Vance et al., 2008); that is, sometimes people may not be aware of their non-social cognitive impairments or may perceive they have cognitive impairments when in fact, they are performing normally. From a social cognitive perspective, if one is experiencing such non-social cognitive impairments, this could hinder their ability to pick up on social cues. On the other hand, if one is worried about having such non-social cognitive impairments, he or she may be uncomfortable being around others or concerned about keeping up with conversations, which could lead to social withdraw. It is unclear from the literature how both objective and subjective non-social cognition interact to affect social cognition. Moving forward, to gain a better understanding of these potential relationships, researchers should consider including both an objective non-social cognitive performance battery as well as a subjective non-social cognitive battery into their protocols.

### Neurological Measures

Neuroimaging or other psychophysical measures (i.e., MRI) were conducted in only five studies. Yet of those, all found significant connections with social cognition. As these studies examined the relationship between brain structure and social cognitive ability, it remains unknown whether functional abnormalities in the brain are related to social cognitive performance in PLHIV. Future studies with functional magnetic resonance imaging [fMRI] may provide valuable information about the neural mechanisms related to social cognitive processes in PLHIV. Only two studies included biomarkers in their study protocols. As certain biomarkers are related to non-social cognition, it is likely many have important associations with social cognition. Rubin et al. (2022a, b) observed that the myeloid migration (MCP-1/MMP-9) factor was associated with reduced facial emotion perception accuracy. Furthermore, instead of examining a standard panel of biomarkers and examining their relationship with social cognition, Kamkwalala et al. (2021) used a novel mechanistic probe (i.e., 10 mg of hydrocortisone) to examine its effect on social cognition, specifically facial emotion recognition/perception. This novel approach implicated a role for the HPA axis in social cognition. The field can advance by examining such biomarkers in studies focusing on social cognition.

### Social Everyday Function

From a practical implication perspective, the evaluation of such social cognitive deficits on social everyday functioning (i.e., isolation, building supportive social networks) remains at the center of this research. Studies in social cognitive research indicate that deficits in social cognition impact social network size, loneliness, and isolation (Beaudoin & Beauchamp, 2020; Eramudugolla et al., 2022). Two studies in this systematic review examined such social everyday outcomes. Clark et al. (2010) found that poorer facial emotion recognition of anger was significantly associated with more distress in not being able to maintain a sense of social connectedness. More specifically, Clark et al. (2010) showed that difficulty in identifying anger was moderately related to self-reported difficulties in intimate social relationships. Similarly, Grabyan et al. (2018) observed that poorer accuracy of facial emotion recognition/perception predicted social cognitive ability. These promising findings indicate that it will be important to examine the relationship between social cognitive ability and social functioning in a systematic way. For instance, it will be important to separate objective social connection (e.g., the number of social relationships, the extent of social networks) from subjective social connection (e.g., loneliness). It will be also important to include other key determinants (e.g., depression) to map the pathways between non-social cognitive ability and social cognition functioning, which could identify the most promising targets for interventions.

### Future Research Implications

Several important future research implications were provided specifically above to strengthen the rigor (i.e., increasing sample size) and direction (e.g., longitudinally designed studies to assess changes in social cognition over time) in this area. Albeit, others require further consideration. First, within this systematic/meta-analysis article, there is an implicit assumption that the neurophysiology of HIV or the psychosocial dynamics of experiencing HIV compromise social cognitive abilities. Yet, it is also likely that premorbid social cognitive deficits may have contributed to one’s HIV infection, and thus the sample of PLHIV may already be a group predisposed to such social cognitive deficits. Unfortunately, methodologically disentangling this requires assessing social cognitive abilities before and after being infected and diagnosed with HIV. Obviously, there is not a simple way to collect such data as it would require administering such social cognitive tests to a large sample of people and wait to see which ones become infected with HIV, and then follow up to see if social cognition changes. Yet, one way to do this would be to employ such social cognitive assessments in large cohort studies such as the MWCCS (Multicenter AIDS Cohort Study/Women’s Interagency HIV Study—Combined Cohort Study) which longitudinally follows large numbers of men and women with HIV and at-risk for HIV. The advantage of the MWCCS studies is that they also gather data on non-social cognition and social everyday functioning (Vance et al., 2016).

A second direction worth further exploration is intervention studies. Intervention studies were not identified in this systematic review; despite some deficits observed in PLHIV, no studies provided an intervention to target deficits in social cognition. Perhaps with the focus on social cognition in the context of HIV infection being a relatively untapped vector of research, it is unclear how profound and/or impactful such deficits in social cognition are to warrant an intervention. If such an intervention was to be provided, three treatment vectors could be examined. First, some evidence suggests that social cognition is more dependent on non-social cognition; for example, González-Baeza et al. (2017) found that vocal prosody was significantly worse in those with HAND than controls, while those PLHIV with normal cognitive functioning performed similarly to controls. Thus, interventions that improve non-social cognition, such as computerized non-social cognitive training (e.g., speed of processing training; Vance et al., 2019), could potentially improve aspects of social cognition as well. Second, social cognitive training programs have been administered to individuals with schizophrenia with some moderate improvements observed (Horan et al., 2018; Lindenmayer et al., 2018; Nahum et al., 2021). As such an approach could be adapted for PLHIV, it will be important to systematically examine the pathways that social cognitive processes affect daily functioning and health-related outcomes in PLHIV. It is possible that social cognitive processes may affect health-related outcomes independent of non-social cognitive impairments. It is also possible that social cognition moderates the relationships between other important factors (i.e., communication with healthcare providers) and health-related outcomes. And third, based on the work by Kamkwalala et al. (2021) and Rubin et al. (2022a, b), low dose hydrocortisone over a 28-day period may be a way to improve social cognition as well as non-social cognition in PLHIV (NCT03237689; R01MH113512).

Thirdly, another area of research is to catalog and map the neuroanatomical substrates and hormonal substrates required for normal social cognition, and then examine how HIV compromises each of these neuroanatomical and hormonal substrates. To do so, one would have to examine this for each type of social cognition (i.e., prosody, empathy, theory of mind, emotional face recognition/perception). For example, clearly prosody which requires auditory processing would require different neuroanatomical and hormonal substrates than emotional face recognition/perception which requires visual processing. For example, the anterior cingulate cortex (ACC) is associated with emotional face recognition/perception, but the ACC is known to be differentially affected in PLHIV; in fact, Clark et al. (2015) found that reduced ACC volume in PLHIW was correlated with fear recognition impairments. Such cataloging of these substrates from the existing literature will also be needed to advance the neuroHIV social cognition literature.

And finally, HIV medications may have a direct or indirect impact on social cognition. It is well-known that many of the legacy HIV medications can have a detrimental impact on brain health and cognition (Dumond et al., 2020; Spence et al., 2022); although these social cognitive HIV studies did not investigate this, it is conceivable that these medications could have negatively impacted social cognition as well. Similarly, many of these legacy HIV medications were also known to have severe side effects (i.e., diarrhea, chronic fatigue, abdominal distress) that could impact one’s ability to socialize; thus, indirectly they could have a dynamic impact on social cognition as well. Moving forward, studies in this area should consider the role in which HIV medications, especially the legacy medications, exert on social cognition.

## Theoretical Implications

**Fig. 4:**
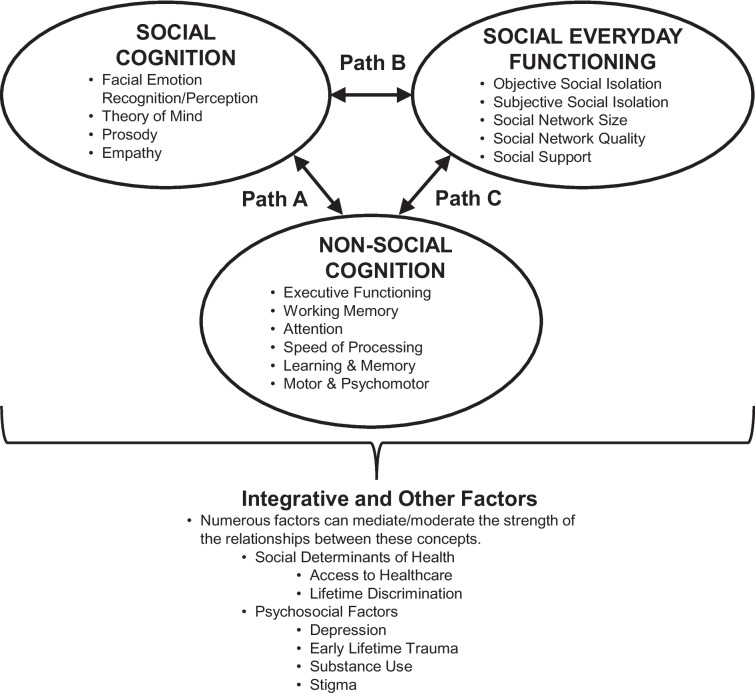
Conceptual framework of social cognition within the context of HIV

From this systematic review and meta-analysis, a rudimentary framework in which to conceptualize the major concepts is proposed in which social cognition is embedded in the larger HIV literature (Fig. 4). We posit that for the field of social cognition to develop in this clinical population, we must examine the interrelationships between social cognition, non-social cognition, and social everyday functioning. Social everyday functioning serves as the target outcome. Social network size is part of the objective social isolation and social network quality is part of the subjective social isolation. Social support can be viewed as part of subjective social isolation. This proposed framework is by no means exhaustive, but it provides a starting point to encourage further discussions and research in this area for PLHIV.

### Social Cognition and Non-Social Cognition (Path A)

From the larger social cognition field, the literature shows a mild to moderate connection between social cognition and non-social cognition (Kubota et al., 2022; Seo et al., 2020). For example, in a sample of 131 adults with early onset schizophrenia, Kubota et al. (2022) found that higher scores on theory of mind was significantly related to higher scores on verbal fluency in women, while higher scores on theory of mind was significantly related to higher scores on executive functioning in men. This relationship has been observed in other measures of social cognition such as facial emotion recognition/perception (Seo et al., 2020).

As shown in our systematic review among studies including PLHIV, the relationship between social cognition and non-social cognition is clearly mixed; in fact, some of these findings may be confounded by the task itself (i.e., emotional processing tasks required quick response time). Yet, given these limitations, of the studies that assessed non-social cognition, cautiously most (7 out of 10) found a mild to moderate association (e.g., Clark et al., 2010; Grabyan et al., 2018; Heilman et al., 2013; Lane et al., 2012). This suggests some of the same neural circuitry for processing social cognition may overlap as non-social cognitive processing, and/or such social cognition is partially dependent on non-social cognitive processing. Thus, as the brain is impacted by HIV pathogenesis or other factors (i.e., drug use, depression, trauma, aging), both social cognition and non-social cognition are compromised together. Yet, as nearly 44% of PLHIV experience HAND (Wei et al., 2020) and with the aging of the HIV population, concerns mount that non-social cognition will be increasingly compromised by age-related comorbidities (Waldrop et al., 2021), as such social cognition may also be partially compromised.

### Social Cognition and Social Everyday Functioning (Path B)

Social cognition plays a key role in social everyday functioning of various populations. Better social cognition is related to better social integration of people in general (Stiller & Dunbar, 2007), and particularly among individuals with alcohol use disorder (Lewis et al., 2019), and individuals with dementia (Eramudugolla et al., 2022). Better social cognition predicts better daily functioning, beyond what has been explained by non-social cognition (Fett et al., 2011; Hoertnagl et al., 2011). Social cognition also mediates the relationship between non-social cognition and daily functioning in individuals with severe mental illness, suggesting that social cognition is a proximal target for improving daily functioning. While most studies on the role of social cognition in daily functioning focus on social integration (i.e., social isolation), emerging evidence from research among people living with substance use disorder suggests the role of social cognition in treatment adherence. Impaired social cognitive performance of individuals with alcohol use disorder was related to poor treatment adherence (Foisy et al., 2007; Rupp et al., 2017).

In this systematic review among studies including PLHIV, only two studies examined the impact of social cognition on social everyday functioning (Clark et al., 2010; Grabyan et al., 2018). Both found that facial emotion recognition/perception was related to (1) a sense of social connectedness, and (2) social ability. Moving forward, HIV studies that examine social everyday functioning should also include outcomes relevant in which social cognition would be critical, such as negotiating interactions with clinicians, medication adherence, and adherence to clinic appointments.

### Non-Social Cognition and Social Everyday Functioning (Path C)

Studies show that a strong relationship exists between non-social cognition and social everyday functioning (i.e., perceived isolation, social network size) (Nie et al., 2021). For example, in a Spanish nationally representative sample of older adults (50 + ; *N* = 1691), Lara et al. (2019) found that over a 3-year period, decreased non-social cognitive function was associated with both social isolation and loneliness. However, as discussed earlier, decreased social interaction may likewise impact non-social cognitive health (Cacioppo & Cacioppo, 2014; Noonan et al., 2018). The non-social cognitive aging literature is filled with such examples (Kelly et al., 2017). Unfortunately, the HIV literature holds very few examples. In a sample of 370 Black and White older adults living with and without HIV, Han et al. (2017) found that poorer non-social cognitive functioning was related to greater loneliness among older Black adults living with HIV. Although more HIV research is needed on this topic, based on the larger literature, it seems likely that there is a dynamic reciprocal relationship between non-social cognition and social everyday functioning among PLHIV.

### Integration and Other Factors

The conceptual framework proposed (Fig. 4) links the main concepts of social cognition, non-social cognition, and social everyday functioning. It is important to note that there are integrative and other factors that may have contributed to being infected with HIV that also impact the relationships between these three concepts. Clearly, the social determinants of health (i.e., access to healthcare, lifetime discrimination) and psychosocial factors (i.e., depression, early lifetime trauma, substance use, stigma) can mediate/moderate the relationships between these key concepts (Halpin et al., 2020; Thompson et al., 2022; Vance, 2013). For instance, the presence or absence of early trauma (Rubin et al., 2022a, b), meth use (Homer et al., 2013), or any of these other factors can impact performance on these social cognition tests. In the context of HIV infection, these social determinants of health and psychosocial factors have been shown to play an important role in all aspects of life including overall health, quality of life, and mortality. For example, depression alone, with a prevalence of 31% in PLHIV (Rezaei et al., 2019), can clearly impact non-social cognition (Waldrop et al., 2021) and interact with social cognition and social everyday functioning. As our knowledge of these relationships emerges between these three basic concepts, this framework will need to be challenged and expanded to incorporate other clinically relevant factors.

## Conclusion

Collectively, based on our systematic review and meta-analysis, these studies suggest that some PLHIV, especially if they have some non-social cognitive impairments, experience subclinical deficiencies in understanding, processing, or describing social stimuli. Such poorer social cognition could lead to more social isolation and decreased social support. As this is an emerging area of HIV research, it is not surprising that there are not social cognitive interventions developed yet.

## Supplementary Information

Below is the link to the electronic supplementary material.

Supplementary file1 (PDF 63 KB)

Supplementary file2 (DOCX 34 KB)

## Data Availability

This is a systematic review. We have provided the search parameters and mesh terms (Table 1) whereby others can replicate the search and reproduce the findings. Actual tabulation of the reviewed articles and summaries of the articles are provided in this systematic review in Tables 2 and 3.

## Appendix: Summary of Social Cognition in HIV Articles

### Baldonero et al. (2013)—Facial Emotion Recognition/Perception

In a two-group cross-sectional study, Baldonero et al. (2013) examined whether deficits in facial emotion recognition (i.e., Ekman and Friesen photographs of anger, disgust, fear, happiness, surprise, and sadness; Young et al., 2002) were observed in 49 PLHIV (*M*_age_ = 49 years) and 20 adults living without HIV (*M*_age_ = 48.5 years). Participants were administered the Facial Emotion Recognition Test (Young et al., 2002), a depression measure, and a neuropsychological test battery of 6 non-social cognitive domains. Compared to those living without HIV, PLHIV performed worse on recognizing fear, even after controlling for age and education; however, group differences in recognizing other emotion-laden facial expressions were not detected including anger, disgust, happiness, surprise, and sadness. Comparing PLHIV with (28.6%) and without (65.3%) non-social cognitive impairment, there were no group differences on fear recognition, suggesting impaired fear recognition cannot be explained by non-social cognitive impairment alone; compared separately, the PLHIV groups with or without non-social cognitive impairment still performed worse than the controls. Yet, poorer verbal recall/memory was related to poor fear recognition. Similarly, poorer overall non-social cognitive ability and more AIDS-defining conditions was related to poorer recognition of happiness. These researchers conclude that impaired fear recognition may serve as a bellwether for poor non-social cognitive functioning. A strength of this study was utilizing an HIV-negative comparison group. Although this study used validated measures of facial emotion recognition and non-social cognition, the sample size was small which limits generalizability.

### Clark et al. (2010)—Facial Emotion Recognition/Perception

In a two-group cross-sectional study, Clark et al. (2010) examined facial emotion recognition ability in 50 PLHIV (*M*_age_ = 46.2 years) and 50 adults living without HIV (*M*_age_ = 44.3 years). Participants were administered a Facial Emotion Recognition Test (i.e., 84 Ekman and Friesen photographs assessing 6 emotional categories; Ekman & Friesen, 1976), a landscape categorization control task (i.e., 84 black-and-white photographs of 6 landscape categories such as tropical), the Benton Test of Facial Recognition (non-emotional face perception abilities), the Inventory of Interpersonal Problems (IIP; Horowitz et al., 1988) which has 8 subscales that assess levels of distress related to interpersonal interaction, and the WTAR (Weschler Test of Adult Reading)—a measure of intelligence. The groups did not differ on their ability to recognize novel faces or being able to categorize pictures of different landscapes; however, PLHIV performed significantly worse overall on recognizing facial emotions. Specifically, PLHIV were significantly less accurate in recognizing fear; however, no significant group differences emerged for the other facial emotions (i.e., angry, disgust, happy, sad, surprise). Since there were no other differences in the other visual recognition tests, this suggests that PLHIV experience a unique deficit in processing emotional-laden information, regarding facial expressions. Furthermore, PLHIV reported significantly more overall distress, including significantly more distress on (1) managing anger and irritability in interpersonal relationships, (2) self-sacrificing behaviors, and (3) desire to connect with others. Interestingly, in post hoc analyses, poorer facial recognition of anger was significantly associated with more distress in maintaining a sense of social connectedness; furthermore, overall intelligence accounted for 23% of the variance in anger recognition accuracy. This study is unique in attempting to measure the impact of social cognition (i.e., facial emotion recognition/perception) with social functioning (i.e., interpersonal distress). Another strength of this of this study was utilizing an HIV-negative comparison group. A limitation of this study was that it had a small sample size.

### Clark et al. (2015)—Facial Emotion Recognition/Perception

In a two-group cross-sectional MRI study, Clark et al. (2015) examined whether regional brain volumes were related to facial emotion recognition/perception abilities in 44 PLHIV (*M*_age_ = 46.4 years) and 44 adults living without HIV (*M*_age_ = 44.3 years); this was a subset of the original study above (Clark et al., 2010). Participants were administered the Benton Test of Facial Recognition, a facial emotion recognition/perception test (i.e., 84 Ekman and Friesen photographs (Ekman & Friesen, 1976)), and emotion neutral faces; MRIs were conducted once within two weeks of administration of these tests. Compared to those living without HIV, PLHIV had larger amygdala and smaller anterior cingulate cortex (ACC) volumes as well as greater impairments in recognizing fearful expression. In PLHIV, reduced ACC volume, but not amygdala volume, was correlated with fear recognition impairments; these findings are the first to show that facial emotion recognition impairments are not associated with HIV-related amygdala enlargements. Group differences in recognizing other emotion-laden facial expressions were not detected; however, the sample size is smaller from Clark et al. (2010) where they did find significant differences. Overall, these findings suggest that as HIV is known to detrimentally impact ACC, such impairments may impact recognizing emotional expressions from others, specifically fear, which could impair social functioning. This study is innovative in that it attempted to link brain imaging data with surrogate measures of social functioning, specifically facial emotion recognition/perception. A strength of this study was utilizing an HIV-negative comparison group. A limitation of this study was that it had a small sample size (i.e., a subset of the original study (Clark et al., 2010)) and the use of only structural neuroimaging data.

### González-Baeza et al. (2016)—Facial Emotion Recognition/Perception

In a two-group cross-sectional study, González-Baeza et al. (2016) examined whether deficits in facial emotion recognition/perception deficits were observed in 107 aviremic PLHIV (*M*_age_ = 47.4 years) and 40 adults living without HIV (*M*_age_ = 42.5 years). Participants were administered the Florida Affect Battery (Bowers et al., 1991) which measures (1) facial identity discrimination, (2) facial affect discrimination, (3) recall of the model’s face, (4) recall of the model’s facial expression, (5) facial affect naming, and (6) facial affect selection. Participants were also administered a neuropsychological test battery that measured seven non-social cognitive domains. Compared to people living without HIV, PLHIV performed worse on recognizing the facial emotion of sadness, even though group differences were not observed in the ability to recognize or recall faces. Similarly, PLHIV performed worse on the facial affect selection task including sadness, anger, and fear. In PLHIV, 24.3% met the Frascati criteria for cognitive impairment. Comparing those PLHIV with and living without cognitive impairment, no group differences emerged between them, except for a trend suggesting that those with cognitive impairment experience poorer facial affect naming. This study suggests that facial emotional processing is compromised in PLHIV; however, non-social cognition is not a strong predictor of such a social cognitive deficit. Another strength of this study was employing an HIV-negative comparison group; however, a limitation of this study was that it utilized a small sample size.

### González-Baeza et al. (2017)—Prosody

In a 2-group cross-sectional MRI study, González-Baeza et al. (2017) examined vocal emotional processing (i.e., vocal prosody) and their correlates to structural brain volumes in 100 PLHIV (*M*_age_ = 47.5 years) and 46 healthy adults without HIV (*M*_age_ = 45.5 years). In addition to a neuropsychological test battery of eight non-social cognitive measures, a vocal prosody matching test was administered. Based on the Florida Affect Battery ninth subtest, this prosody test required participants to listen to nine different auditory sentences that were emotionally-ladened (i.e., sadness, anger, fear, happiness, and neutral). Participants were required to listen to these sentences and match them emotionally to faces expressing the same emotion. MRI scans were conducted on a subset of 36 PLHIV. Higher vocal emotional processing scores were associated with larger brain volumes in the following regions: left hippocampus, right frontal, bilateral thalamus, and temporal and parietal lobes. Most of these associations remained after controlling for neuropsychological test performance of non-social cognition (i.e., global composite). Compared to people living without HIV, PLHIV performed worse on vocal prosody. Notably, the individuals driving this difference were those PLHIV with HIV-Associated Neurocognitive Disorder (HAND). These findings suggest that non-social cognitive functioning supports vocal emotional processing; unfortunately, it is not clear what cognitive domain is utilized in processing emotional content in sound. Strengths of this study included utilizing an HIV-negative comparison group, a large sample size, and measures of MRI and a neuropsychological test battery of non-social cognition. A weakness of this study includes only the use of structural neuroimaging data to examine neural abnormalities related to prosody perception.

### Grabyan et al. (2018)—Facial Emotion Recognition/Perception

In a three-group cross-sectional study, Grabyan et al. (2018) examined whether PLHIV demonstrating cognitive impairment (*n* = 37; *M*_age_ = 43.95 years) would perform poorer on an emotional processing task, as measured by facial affect discrimination, compared to PLHIV without HAND (*n* = 46; *M*_age_ = 46.59 years) and adults living without HIV (*n* = 38; *M*_age_ = 44.3 years). Participants were administered the CogState Social Emotional Cognition Task (SECT) and the UCSD Performance-based Skills Assessment-Brief (UPSA-B). The SECT consists of 48 trials; in each trial, participants were shown four sets of eyes or computerized faces at once on a computer screen that convey different emotions or intensity of emotions for 15 s. Participants were instructed to select the image that is different, no other instructions were provided. The computer program scores according to accuracy (i.e., age norms applied) and speed of response. The UPSA-B required participants to role-play in completing real world tasks, which includes a communication subscale (e.g., a telephone task) and a financial subscale (e.g., paying a bill).

Compared to PLHIV without HAND, those with HAND were 10 times more likely to be impaired on emotional processing accuracy. Among PLHIV with HAND, poorer memory was associated with poorer SECT accuracy. Additionally, PLHIV with HAND were slower than both PLHIV without HAND and people living without HIV. Finally, among PLHIV (with and without HAND), lower accuracy was associated with poorer functional capacity (tapping social ability) but not financial capacity. This study demonstrated that emotional processing maybe be disrupted in PLHIV, especially in those with cognitive impairment. Strengths of this study included utilizing a large sample size with groups controlling for HAND and HIV-status, and measures of performance-based social engagement (i.e., UPSA-B communication scale) and of non-social cognition.

### Heilman et al. (2013)—Facial Emotion Recognition/Perception

In a two-group cross-sectional study, Heilman et al. (2013) examined whether the Polyvagal Theory could explain auditory processing and facial emotional recognition/perception in 61 women living with HIV (*M*_age_ = 42.16 years) and 22 women living without HIV (*M*_age_ = 38.09 years). This theory ascribes that HIV disrupts normal autonomic function via the vagus nerve which has corresponding impact on heart rate variability and brain stem activity that regulate auditory processing via the middle ear muscles needed for extracting human speech, pharyngeal and laryngeal muscles for speech, facial muscles needed for emotional expression, and neck muscles needed for gesturing (i.e., tilting). It is suggested that this hear-face connection provides much of the neural mechanisms responsible for the Social Engagement System; a convergence of synergistic somatomotor and visceromotor components that are integrated with social engagement and state regulation processes that help maintain social behavior (Porges, 2007). Participants wore a Lifeshirt during their study visit; Lifeshirt is a device that measures heart rate and respiratory sinus arrhythmia. Participants were also administered two measures of auditory processing (i.e., Filtered Words and Competing Words), and a facial emotion recognition/perception test (i.e., Dynamic Affect Recognition Evaluation—DARE) (Porges, 2007) and the WTAR (Weschler Test of Adult Reading)—a measure of intelligence. Filtered Words assessed the ability to listen to words when they are muffled and approximate the ability to distinguish human speech. Competing Words assessed dichotic listening skills; specifically, participants were instructed to correctly report the word being presented to either the left or right ear when two different words are being presented separately to the left and right ear simultaneously. DARE, a dynamic standardized tool for measuring facial emotion recognition/perception, entails the presentation of neutral faces that gradually morph to one of six targeted emotional expressions (i.e., happiness, anger, fear, disgust, surprise, fear, sad). During these trials, participants were instructed to indicate as soon as they recognized the emotion (latency) and verbally identify the emotion (accuracy). Researchers found several significant patterns. First, respiratory sinus arrhythmia was lower in women living with HIV. Second, women living with HIV performed worse on the Competing Words test, and although not significant, they also performed worse on the Filtered Words test. Third, women living with HIV performed worse on the accuracy of the DARE test, although there were no group differences on any specific emotion nor were there differences in the latency of the DARE test. Fourth, across both women living with and without HIV, greater intelligence (WRAT) was related to fewer accuracy errors on DARE sad, disgust, anger, and total videos. In women living with HIV, greater intelligence was related to fewer accuracy errors on DARE across all videos and the anger videos, which could suggest that non-social cognitive function supports social cognition. Heilman et al. (2013) concluded that their data supported the hypothesis, that is, HIV detrimentally impacts vagal tone which reduced respiratory sinus arrhythmia that in turn reduced dichotic listening abilities and facial emotional recognition that contributes to poorer functioning of the social engagement system, potentially compromising social cognition and social functioning. This study was unique in that it examined Social Engagement Theory with heart rate variability (via respiratory sinus arrhythmia) to examine facial emotion recognition/perception. Other study strengths included utilizing an HIV-negative control group and a measure of non-social cognition (i.e., WRAT, although it lacked a full non-social neuropsychological test battery); however, limitations included a small sample size and the auditory task lacked emotional valence.

### Homer et al. (2013)—Theory of Mind & Facial Emotional Recognition/Perception

In a four-group, cross-sectional study of 56 men who have sex with men (MSM) (*M*_age_ = 40 years; *SD* = 10.6 years; range = 19–70), participants were compared on two measures of social cognition across HIV serostatus (32 PLHIV & 24 HIV-uninfected individuals) and methamphetamine use (29 users & 27 non-users) to form four groups: (1) HIV-negative with methamphetamine use (*n* = 16), (2) PLHIV with methamphetamine use (*n* = 13), (3) HIV-negatives with no methamphetamine use (*n* = 8), and (4) PLHIV with no methamphetamine use (*n* = 19). In addition to a measure of executive function (i.e., the Stroop Color and Word Test), two social cognitive measures were administered: (1) Faux Pas Recognition Task (Faux Pas Task, a theory of mind test) and (2) Reading the Mind in the Eyes Task (Eyes Task; a facial emotion recognition/perception test; Baron-Cohn et al., 2001). In the Faux Pas Task, participants were told 20 brief stories, half control and half with a faux pas. The faux pas stories contained an element in which a character said something socially awkward or inappropriate. Participants received a point for each story in which they correctly identified whether a faux pas occurred, and they received another point if they provided a rationale for why something was socially awkward or inappropriate; a maximum score of 30 was possible. In the Eyes Task, participants looked at 36 faces and used a list of words to describe the emotions expressed in each face (e.g., panicked, hateful, arrogant, and jealous). Responses were scored as incorrect (0) or correct (1); scores ranged from 0 to 36.

Researchers found that with the Faux Pas Task, methamphetamine users performed worse than nonusers (*p* = 0.03, partial *η*^*2*^ = 0.08). Although PLHIV performed worse on the Faux Pas Task than those living without HIV, this difference was not significant (*p* = 0.28, *d* =  − 0.45). Likewise, the interaction between methamphetamine use and HIV status was not significant (*p* = 0.71, partial *η*^*2*^ = 0.00). In the Eyes Task, methamphetamine users performed worse on the Eyes Task than nonusers (*p* = 0.004, partial *η*^*2*^ = 0.15); similarly, PLHIV performed worse on the Eyes Task than those without HIV (*p* = 0.02, partial *η*^*2*^ = 0.10); however, the association between methamphetamine use and performance was not moderated by HIV-serostatus (*p* = 0.38, *d* = 0.24). In general, these combined results suggest that methamphetamine users had more social cognitive impairment than nonusers as exhibited by lower scores on the Eyes Task and the Faux Pas Task, and that compared to those living without HIV, PLHIV had more social cognitive impairment on the Eyes Task. These researchers suggested that both methamphetamine use and HIV infection have been shown to exert additive or perhaps interactive damage to the prefrontal cortex (Isper et al., 2015; Tehrani et al., 2019) which may compromise social cognition; however, the executive function test was not related to social cognitive performance. A strength of this study is the use of established measures of social cognition, while limitations of this study include the small sample size, the unspecified amount of methamphetamine use, and the lack of a full non-social neuropsychological test battery. In fact, it has been suggested that individuals who engage in sexual risk behaviors leading to HIV infection or those who engage in drug use may already have poor non-social cognitive functioning to begin with already, which after HIV infection and further drug use further compromises non-social cognition as well as social cognition.

### Kamkwalala et al. (2021)—Facial Emotion Recognition/Perception

In a two-group, double-blind, placebo-controlled, cross-over study of 31 women living with HIV (*M*_age_ = 36.40 years) and 34 men living with HIV (*M*_age_ = 31.85 years), Kamkwalala et al. (2021) used as a mechanistic probe (i.e., single low-dose of hydrocortisone (10 mg, LDH) vs. placebo) to explore sex differences on the effects of glucocorticoids on negative emotion perception. The Facial Emotion Perception Test (FEPT; Langenecker et al., 2005) was used. The FEPT is a computerized task that presents pictures of animals and people in which participants rate the emotional valence of each picture being observed. Accuracy in identifying emotional expression (i.e., sadness, fear, anger, happiness, neutral) served as the primary outcome; reaction time serves as a secondary outcome. Stress/anxiety, childhood trauma, and salivary cortisol levels were also measured.

Accuracy in FEPT, especially for fearful faces, was influenced by LDH administration in women only. This threat detection, sensitivity to detect fearful faces, was significant in women with lower overall childhood trauma (*r* =  − 0.40, *p* = 0.02), lower overall childhood emotional abuse (*r* =  − 0.35, *p* = 0.05), lower overall childhood physical neglect (*r* =  − 0.43, *p* = 0.01), lower overall childhood physical abuse (*r* =  − 0.49, *p* = 0.005), and lower overall anxiety (*r* =  − 0.32, *p* = 0.08). These findings suggest that women with more severe trauma have a blunting of this LDH effect of increased sensitivity to fearful face recognition. This finding suggests that women living with HIV without trauma have heightened sensitivity to fearful faces; perhaps they also have heightened sensitivity to other fear-provoking social cues. It also suggests a role of the HPA axis and glucocorticoid receptor activity/availability in improving neural signals from the emotion processing and facial processing brain regions, especially in women. Furthermore, the relationships to the accuracy of identifying fearful faces with non-social cognitive measures were mixed, with some correlations showing better non-social cognitive outcomes (i.e., working memory of Letter-Number-Sequence) and some showing worse non-social cognitive functioning (i.e., Trails A and B). This study is one of the few that examined early life trauma, and the only one that examined sex differences and performed a pharmacologic challenge to investigate low dose hydrocortisone to examine sex differences in facial emotion recognition/perception; despite a small sample size, the data gathered from such an experimental study is novel.

### Lane et al. (2012)—Facial Emotion Recognition/Perception

In a two-group cross-sectional study, Lane et al. (2012) examined whether facial emotion recognition/perception was compromised in PLHIV. To measure facial emotional processing, 85 PLHIV (*M*_*age*_ = 53.68 yrs) and 25 adults living without HIV (*M*_age_ = 55.40 years) were administered the Penn Emotion Recognition Task (i.e., identifying the emotion of a face), Penn Emotion Discrimination Task (i.e., determine whether two faces express the same emotional intensity), and Penn Emotional Acuity Test (i.e., determine emotional value of a face; Gur et al., 2001). In addition, participants were administered a standardized non-social neuropsychological test battery to determine HAND, measures of depressive symptomatology, and biomedical measures (i.e., CSF HIV RNA, plasma HIV RNA).

In general, subtle differences on facial emotional processing were observed. Compared to those living without HIV, PLHIV were slower to recognize fearful faces, and they were poorer in discriminating intensity of happy expressions and identifying sad expressions. Compared to PLHIV without HAND, PLHIV with HAND had (1) slower recognition of happiness, anger, and sadness, and (2) poorer discrimination of happiness. Among PLHIV, poorer overall non-social cognitive performance was associated with poorer discrimination of happiness (*R*^*2*^ = 0.22, *p* < 0.001). Moreover, slower reaction time of fear was associated with poorer performance in speed of information processing, motor functioning, and mental flexibility, while poorer performance in speed of information processing, motor functioning, and mental flexibility were associated with poorer happy faces discrimination. Unfortunately, this study was limited by numerous comparisons making it vulnerable to Type I errors. Yet, the strengths of this study included the use of an age-comparable HIV-negative control group, although it was a small group which likely reduced power.

### Lysaker et al. (2012)—Theory of Mind & Facial Emotion Recognition/Perception

In a two-group cross-sectional study, Lysaker et al. (2012) examined metacognition and social functioning (i.e., Theory of Mind & Facial Emotion Recognition/Perception) between 40 adults with schizophrenia (*M*_age_ = 48.84 years) and 25 PLHIV (*M*_age_ = 51.68 years). Researchers hypothesized that deficits in social cognition and metacognition observed in schizophrenia may be caused by their medical and social adversity; to account for this adversity, PLHIV were chosen as a control group since they have historically experienced such adversity. Participants were administered the Metacognition Assessment Scale, the Bell-Lysaker Emotional Recognition Task (BLERT), the Hinting test, and the Hopkins Verbal Learning Test. The Metacognition Assessment Scale subjectively assesses the following subscales: self-reflectivity, understanding of other’s mind, decentration (i.e., ability to see oneself in a larger environment from numerous perspectives), and mastery (i.e., ability to understand mental states to solve problems). The BLERT, a measure of facial emotion recognition/perception, requires one to view video segments of an actor and identify one neutral, two positive, and four negative emotional states; scores range from 0 to 21 with higher scores indicative of better social cognition. The Hinting test, a measure of theory of mind, consists of the participant reading a story in which he/she must pick up the “hints” as to the intention of the fictional characters.

Overall, individuals with schizophrenia performed significantly worse on the Hinting test and all the MAS subscales. Interestingly, both groups were not different on the BLERT (schizophrenia *M* = 12.29, *SD* = 3.25; PLHIV *M* = 13.60, *SD* = 2.69); this suggests that PLHIV may be functioning at a similar level with those with schizophrenia who have difficulty recognizing or processing facial affect. Lysaker et al. (2012) speculate that the causes of poorer processing of facial affect may be different in PLHIV than those with schizophrenia. They suggest that perhaps (1) PLHIV may have attributional biases due to their disease status (e.g., ascribing disgust or anger to faces because of internalized stigma and fear of being rejected), and (2) some PLHIV may have had difficulty with interpreting the emotional valence of faces which may have been a contributing factor for contracting HIV. One limitation of this study is that the concept of adversity is amorphous, and it cannot be assumed that the level of adversity is equivalent between these two groups. Another limitation is that there is not a clear normative comparison group; thus, for the purposes of this systematic review, it is not clear how PLHIV compare to controls. Interestingly, although there are no norms for the BLERT, Pinkham et al. (2018) found in 154 healthy controls, participants scored on average 15.92 (*SD* = 2.70) on the BLERT. This suggests that PLHIV were performing slightly worse (*M* = 13.60) and those with schizophrenia (*M* = 12.29) slightly more worse than healthy controls, but not dramatically. Although the sample size is small which limits generalizability, this study is innovative in that it provides a comparison of PLHIV to a clinical population known for impaired social cognition.

### Lysaker et al. (2014)—Facial Emotion Recognition/Perception

In a two-group cross-sectional study, controlling for an index of social cognition, Lysaker et al. (2014) examined metacognitive capacity between 166 individuals living with schizophrenia (*M*_age_ = 48.4 years) and 57 PLHIV (*M*_age_ = 48.3 years). Researchers examined whether metacognitive capacity is greater in those with schizophrenia after controlling for social cognition; however, they wanted to control for this in a sample that also experiences medical adversity but without psychosis, thus PLHIV were chosen. Their index for social cognition was the BLERT, a measure of facial emotion recognition/perception. The BLERT requires one to view video segments of an actor and identify one neutral, two positive, and four negative emotional states; scores range from 0 to 21 with higher scores indicative of better social cognition. Other measures pertinent to their study were not relevant for this systematic review as it really focused on metacognition, and only minimally focused on social cognitive differences.

Overall, on the BLERT, individuals living with schizophrenia performed slightly worse (*M* = 12.8; *SD* = 0.3) than PLHIV (*M* = 14.1, *SD* = 0.4) but the two groups were not significantly different on the BLERT. These findings suggest that PLHIV may be functioning at a similar level with those with schizophrenia who have difficulty recognizing or processing facial affect. This study has many of the same strengths and limitations as the other Lysaker et al. (2012) study, primarily that it is was not designed to examine social cognition in the context of HIV infection, but instead PLHIV were included as a medical population with significant life adversity without psychosis that served as a type of control for schizophrenia.

### Rubin et al. (2022a, b)—Facial Emotion Recognition/Perception

In a two-group cross-sectional study of secondary data analysis of a placebo group in a pharmacologic challenge study (i.e., Kamkwalala et al., 2021), Rubin et al. (2022a, b) examined the dynamic relationships between early life trauma, social cognition, and inflammation and neuroendocrine factors in 58 PLHIV. Using the Childhood Trauma Questionnaire (i.e., sexual abuse 8 or higher; or physical abuse 13 or higher; or emotional abuse 13 or higher), 50% of the sample endorsed at least moderate early life trauma (*M*_age_ = 32.7 years, *SD* = 9.1 years) and 50% did not (*M*_age_ = 34.6 years, *SD* = 8.7 years). Facial emotion recognition/perception was measured using the Facial Emotion Perception Test (Langenecker et al., 2005). This instrument is a computerized task that presents pictures of animals and people in which participants rate the emotional valence of each picture being observed. Accuracy in identifying emotional expression (i.e., sadness, fear, anger, happiness, neutral) served as the primary outcome; reaction time served as a secondary outcome. Nine cytokines (i.e., TNF-α, IL-6, CRP (C-reactive protein)) were collected through saliva. Neuroendocrine factors, oxytocin (OT) and arginine vasopressin, were assessed via blood using enzyme immunoassay kits. Linear mixed models were used to examine the influence of group, type of facial expression, and interactions, along with covariates (i.e., age, sex, current cannabis use). To reduce the number of tests of each of the 12 neurobiological markers, a principal components analysis revealed 5 factors to be used as predictor variables: factor 1 (1-proinflammatory cytokines), factor 2 (myeloid migration), factor 3 (3-OT/CRP), factor 4 (chemokines regulating immune cell trafficking), and factor 5 (5-HPA axis hormones).

Compared to those without early life trauma, those with early life trauma performed worse overall on the accuracy of Facial Emotion Identification Test (*p* = 0.021, *d* = 0.63); however, a pattern did not emerge as to which types of emotional faces participants had difficulties identifying. Those with early life trauma also had higher levels of oxytocin but not arginine vasopressin (*p* = 0.039, *d* = 0.57). Compared to those without early life trauma, those with early life trauma with low factor 3 (3-OT/CRP) performed worse on the accuracy of the Facial Emotion Test (*p* < 0.05); this pattern was not observed for those who scored higher on factor 3. Those who had higher factor 2 (myeloid migration), regardless of early life trauma, performed worse on total recognition accuracy (β =  − 0.39; *p* = 0.0026) as well as for happy (β =  − 0.33; *p* = 0.017), angry (β =  − 0.32; *p* = 0.018), sad (β =  − 0.32; *p* = 0.022), fearful (β =  − 0.28; *p* = 0.038), and neutral faces (β =  − 0.24; *p* = 0.061). Finally, for those with early life trauma, a trend emerged where those high on factor 5 (HPA axis hormones) performed worse on total recognition accuracy (p = 0.099 for interaction) than those without early life trauma; this trend was not significant for those low on factor 5. These findings suggest that early life trauma can detrimentally impact social cognition; in fact, certain neurobiological processes may modulate such functioning. Oxytocin and C-reactive protein (factor 3) emerged as a possible target for intervention in those with early life trauma, and myeloid migration markers (factor 2) emerged as a target for PLHIV in general. This innovative study took a deeper dive into the neurobiological mechanisms that regulate social cognition, which represents a major study strength. Study limitations include the cross-sectional nature of the study, no seronegative controls, and a small sample with mostly men (72.4%).

### Walzer et al. (2016)—Empathy

In a one-group cross-sectional study, Walzer et al. (2016) examined the association of self-focus and other-focus perspective taking on risky health behaviors in 79 PLHIV (*M*_age_ = 41.86 years). Participants completed questionnaires pertaining to several areas: HIV/AIDS knowledge, exaggerated internal locus of control, and their willingness to engage in 11 HIV/AIDS risk behaviors (i.e., “sharing a needle while shooting up drugs with friends” or “having unprotected oral sex”). Concerning social cognitive variables of being other-focused, two 7-item scales were used to assess other perspective taking or theory of mind (i.e., “I try to look at everybody’s side of a disagreement before I make a decision”) or empathic concern (i.e., “I am often touched by things I see happen”); similarly, a self-focused perspective of personal distress was measured on a similar 7-item scale (i.e., “In emergency situations, I feel apprehensive and ill-at-ease”). Responses ranged from 1 (“does not describe me well”) to 7 (“describes me very well”). As hypothesized, those with more other-focused perspectives indicated that they were less likely to engage in risky behaviors while those with more self-focused perspective reported greater willingness to engage in such risk behaviors. It is thought that the other-focused perspective enhances how one modifies his/her behavior to prevent harm on other people and society. A limitation of this study was that the measure of empathy and theory of mind was measured by a self-report questionnaire; however, Cronbach’s alphas were marginally acceptable (α = 0.59 and 0.60). Although the sample size was small which limits generalizability, this study was innovative in that it provided a unique and direct examination of the theory of mind and empathy in PLHIV.
