# Supplementary material for: A Systematic Review and Meta-Analysis of Social Cognition Among People Living with HIV: Implications for Non-Social Cognition and Social Everyday Functioning
Source: Neuropsychol Rev. 2024 Jun 13;35(2):381–410. doi: 10.1007/s11065-024-09643-5 (PMC12328480; doi:10.1007/s11065-024-09643-5)
Supplement: Supplementary file 1 — Supplementary file1 (PDF 63 KB) [file 11065_2024_9643_MOESM1_ESM.pdf]

A	B	C	D	E	F	G	H	I	J	K	L	M	N	O	P	Q	R	S	T
ID	Title	First Auth	Year	N_PWH	N-Control	Age-PWH	Age-Contr	Task	Task_Long	Task_Num	Measure	Mean_PW	SD_PWH	Mean_Cor	SD_contro	Effect size	es	X	vares
1	Baldonero	Baldonero	2018	49	20	49	48.5	Facial affe	Facial Affe	1	Global sco	50	3.8	48.7	5.2		-0.3063	-0.3063	0.071073
3	Clark (201	Clark	2015	44	44	46.4	44.3	Facial affe	Facial Emc	1	accuracy (	79.2	9	83.1	8.1		0.45551	0.45551	0.046613
4	Gonzalez-	Gonzalez-	2016	107	40	47.4	42.5	Facial affe	Facial Affe	1						0.24		0.24	0.034542
4	Gonzalez-	Gonzalez-	2016	107	40	47.4	42.5	Facial affe	Facial Affe	1						0.05		0.05	0.034354
4	Gonzalez-	Gonzalez-	2016	107	40	47.4	42.5	Facial affe	Facial Disc	1						0.02		0.02	0.034347
4	Gonzalez-	Gonzalez-	2016	107	40	47.4	42.5	Facial affe	Facial Affe	1						0.22		0.22	0.03451
5	Grayban (2	Grayban	2018	37	46	44.3	46.5	Facial affe	MSCET Bra	1	age-correc	49.92	9.08	52.36	5.7		0.329932	0.329932	0.04941
6	Heilman (2	Heilman	2013	55	21	42.16	38.1	Facial affe	Dynamic A	1		76.96	14.15	83.46	12.8		0.471077	0.471077	0.067231
8	Lane (201	Lane	2012	85	25	55.4	53.68	Facial affe	PENN Emc	1	total corre	31.08	3.31	32.12	3.28		0.314831	0.314831	0.052209
11	Gonzalez-	Gonzalez-	2017	100	46	47.5	45.5	Prosody	Prosody pe	2	number o	6.4	1.4	7	1.7		0.399944	0.399944	0.032281
13	Homer (20	Homer	2013	32	24			Theory of	Eyes task	3		21.1	6.8	26.1	4.8		0.829215	0.829215	0.078886
13	Homer (20	Homer	2013	32	24			Theory of	Faux Pas t	3		20.4	5.1	22.4	3.7		0.438924	0.438924	0.074589
