# Supplementary material for: A Systematic Review and Meta-Analysis of Social Cognition Among People Living with HIV: Implications for Non-Social Cognition and Social Everyday Functioning
Source: Neuropsychol Rev. 2024 Jun 13;35(2):381–410. doi: 10.1007/s11065-024-09643-5 (PMC12328480; doi:10.1007/s11065-024-09643-5)
Supplement: Supplementary file 2 — Supplementary file2 (DOCX 34 KB) [file 11065_2024_9643_MOESM2_ESM.docx]

| **Section and Topic** | **Item #** | **Checklist item** | **Location where item is reported** |
| --- | --- | --- | --- |
| **TITLE** | | |  |
| Title | 1 | Identify the report as a systematic review. | Title page (page 1) |
| **ABSTRACT** | | |  |
| Abstract | 2 | See the PRISMA 2020 for Abstracts checklist. | This is the checklist. The Abstract is in the Abstract section (pages 1-2). |
| **INTRODUCTION** | | |  |
| Rationale | 3 | Describe the rationale for the review in the context of existing knowledge. | This is provided in the Introduction of the article (pages 3-7). |
| Objectives | 4 | Provide an explicit statement of the objective(s) or question(s) the review addresses. | This is provided in the Introduction of the article (pages 6-7). |
| **METHODS** | | |  |
| Eligibility criteria | 5 | Specify the inclusion and exclusion criteria for the review and how studies were grouped for the syntheses. | This is provided in the Systematic Review Methodology section (pages 7-9). |
| Information sources | 6 | Specify all databases, registers, websites, organisations, reference lists and other sources searched or consulted to identify studies. Specify the date when each source was last searched or consulted. | This is provided in the Systematic Review Methodology section and in Figure 1 (pages 7-9). |
| Search strategy | 7 | Present the full search strategies for all databases, registers and websites, including any filters and limits used. | This is provided in the Systematic Review Methodology section and in Table 1 (pages 7-9). |
| Selection process | 8 | Specify the methods used to decide whether a study met the inclusion criteria of the review, including how many reviewers screened each record and each report retrieved, whether they worked independently, and if applicable, details of automation tools used in the process. | This is provided in the Systematic Review Methodology section (pages 7-9). |
| Data collection process | 9 | Specify the methods used to collect data from reports, including how many reviewers collected data from each report, whether they worked independently, any processes for obtaining or confirming data from study investigators, and if applicable, details of automation tools used in the process. | This is provided in the Systematic Review Methodology section (pages 7-9). |
| Data items | 10a | List and define all outcomes for which data were sought. Specify whether all results that were compatible with each outcome domain in each study were sought (e.g. for all measures, time points, analyses), and if not, the methods used to decide which results to collect. | This is provided in the Systematic Review Methodology section (page 8). |
|  | 10b | List and define all other variables for which data were sought (e.g. participant and intervention characteristics, funding sources). Describe any assumptions made about any missing or unclear information. | This is provided in the Systematic Review Methodology section (page 8). |
| Study risk of bias assessment | 11 | Specify the methods used to assess risk of bias in the included studies, including details of the tool(s) used, how many reviewers assessed each study and whether they worked independently, and if applicable, details of automation tools used in the process. | This is provided in the Systematic Review Methodology section (page 8). |
| Effect measures | 12 | Specify for each outcome the effect measure(s) (e.g. risk ratio, mean difference) used in the synthesis or presentation of results. | This information is provided in the Meta-Analysis of Social Cognition in the Context of HIV Infection and Figures 3 and 4. |
| Synthesis methods | 13a | Describe the processes used to decide which studies were eligible for each synthesis (e.g. tabulating the study intervention characteristics and comparing against the planned groups for each synthesis (item #5)). | This is provided in the Systematic Review Methodology section (pages 7-9). |
|  | 13b | Describe any methods required to prepare the data for presentation or synthesis, such as handling of missing summary statistics, or data conversions. | For the systematic review, this was not an issue. For the meta-analysis, this was an issue. As such, this is provided in the Systematic Review Methodology section (pages 13-15). |
|  | 13c | Describe any methods used to tabulate or visually display results of individual studies and syntheses. | This is provided in the Systematic Review Methodology section (pages 7-9). Also, Tables 2 and 3 and Figures 3 and 4 were used to tablet and visually display studies and syntheses. |
|  | 13d | Describe any methods used to synthesize results and provide a rationale for the choice(s). If meta-analysis was performed, describe the model(s), method(s) to identify the presence and extent of statistical heterogeneity, and software package(s) used. | This is provided in the Systematic Review Methodology section (pages 7-9) as well as the Systematic Review Methodology section (pages 13-15). |
|  | 13e | Describe any methods used to explore possible causes of heterogeneity among study results (e.g. subgroup analysis, meta-regression). | This is provided in the Systematic Review Methodology section (pages 13-15).  We also provide this information in the Meta-Analysis section. |
|  | 13f | Describe any sensitivity analyses conducted to assess robustness of the synthesized results. | Not applicable |
| Reporting bias assessment | 14 | Describe any methods used to assess risk of bias due to missing results in a synthesis (arising from reporting biases). | Not applicable. We did not have missing results.  However, in Table 2 and the study summaries (Appendix), we did identify strengths/limitations of each study. |
| Certainty assessment | 15 | Describe any methods used to assess certainty (or confidence) in the body of evidence for an outcome. | Not applicable |
| **RESULTS** | | |  |
| Study selection | 16a | Describe the results of the search and selection process, from the number of records identified in the search to the number of studies included in the review, ideally using a flow diagram. | This is provided in Figure 1 and the Systematic Review Methodology section (pages 7-9) |
|  | 16b | Cite studies that might appear to meet the inclusion criteria, but which were excluded, and explain why they were excluded. | This is provided in Figure 1 and the Systematic Review Methodology section (pages 7-9) |
| Study characteristics | 17 | Cite each included study and present its characteristics. | This information can be found in Table 2, Table 3, and the Appendix. |
| Risk of bias in studies | 18 | Present assessments of risk of bias for each included study. | We assessed study strengths/limitations in narratives written for each article and provided this risk of bias in Table 3, Table 4, and the Appendix. The risk of bias normally refers to intervention studies and evaluates methodological factors that pertain to assessing interventions (i.e., allocation concealment (selection bias), blinding of participants); our systematic review is composed of all cross-sectional studies (except for 1 experimental study that is not testing an intervention); thus, these criteria for risk of bias really don’t fit. |
| Results of individual studies | 19 | For all outcomes, present, for each study: (a) summary statistics for each group (where appropriate) and (b) an effect estimate and its precision (e.g. confidence/credible interval), ideally using structured tables or plots. | This is provided in Figure 3, Figure 4, and the Systematic Review Methodology section (pages 13-15). |
| Results of syntheses | 20a | For each synthesis, briefly summarise the characteristics and risk of bias among contributing studies. | This is provided in detail in the section on Systematic Review Synthesis of Findings section (pages 9-3) and the Discussion section (pages 15-20). |
|  | 20b | Present results of all statistical syntheses conducted. If meta-analysis was done, present for each the summary estimate and its precision (e.g. confidence/credible interval) and measures of statistical heterogeneity. If comparing groups, describe the direction of the effect. | This is provided in Figure 3, Figure 4, and the Systematic Review Methodology section (pages 13-15). |
|  | 20c | Present results of all investigations of possible causes of heterogeneity among study results. | This is provided in Figure 3, Figure 4, and the Systematic Review Methodology section (pages 13-15). |
|  | 20d | Present results of all sensitivity analyses conducted to assess the robustness of the synthesized results. | Not applicable |
| Reporting biases | 21 | Present assessments of risk of bias due to missing results (arising from reporting biases) for each synthesis assessed. | Not applicable. We did not have missing results.  However, in Table 2 and the study summaries (Appendix), we did identify strengths/limitations of each study. |
| Certainty of evidence | 22 | Present assessments of certainty (or confidence) in the body of evidence for each outcome assessed. | Not applicable |
| **DISCUSSION** | | |  |
| Discussion | 23a | Provide a general interpretation of the results in the context of other evidence. | This is provided in the Discussion section. |
|  | 23b | Discuss any limitations of the evidence included in the review. | This is provided in the Discussion section. |
|  | 23c | Discuss any limitations of the review processes used. | This is provided in the Discussion section. |
|  | 23d | Discuss implications of the results for practice, policy, and future research. | This is provided in the Discussion section. |
| **OTHER INFORMATION** | | |  |
| Registration and protocol | 24a | Provide registration information for the review, including register name and registration number, or state that the review was not registered. | Not registered; we tried several times but the PROPSERO system would not accept it. Since you can’t contest it, we are no sure why it was not accepted. We suspect it was because we did not have a single outcome variable, but instead had several social cognitive measures that were part of this systematic investigation. |
|  | 24b | Indicate where the review protocol can be accessed, or state that a protocol was not prepared. | This article serves as the review protocol. |
|  | 24c | Describe and explain any amendments to information provided at registration or in the protocol. | Not applicable. |
| Support | 25 | Describe sources of financial or non-financial support for the review, and the role of the funders or sponsors in the review. | Not applicable. |
| Competing interests | 26 | Declare any competing interests of review authors. | None |
| Availability of data, code and other materials | 27 | Report which of the following are publicly available and where they can be found: template data collection forms; data extracted from included studies; data used for all analyses; analytic code; any other materials used in the review. | Not applicable. |

*From:*  Page MJ, McKenzie JE, Bossuyt PM, Boutron I, Hoffmann TC, Mulrow CD, et al. The PRISMA 2020 statement: an updated guideline for reporting systematic reviews. BMJ 2021;372:n71. doi: 10.1136/bmj.n71

For more information, visit: <http://www.prisma-statement.org/>
